# School-based health education for dengue control in Kelantan, Malaysia: Impact on knowledge, attitude and practice

**DOI:** 10.1371/journal.pntd.0008075

**Published:** 2020-03-27

**Authors:** Rattanam AhbiRami, Wan Fatma Zuharah

**Affiliations:** 1 School of Biological Control, Universiti Sains Malaysia, Minden, Penang, Malaysia; 2 Vector Control Research Unit, School of Biological Sciences, Universiti Sains Malaysia, Minden, Penang; The Natural History Museum London, UNITED KINGDOM

## Abstract

The massive flood in Malaysia’s east coast in December 2014 has placed Kelantan in a possible dengue outbreak risk. At this point, community awareness is essential in preventing disease spread. However, no data on knowledge, attitude, and practice (KAP) of dengue in Kelantan have existed in relevance to flood disaster, although such information is necessary for the vector control programs. The purpose of this study is to assess the KAP regarding dengue among school children from flooded and unflooded areas and to evaluate the effectiveness of the dengue health education program in improving their KAP level. A school-based pre- and post-tests design was utilized in this study whereby a booklet on dengue was distributed during the interphase of the tests. The information collected was on the socio-demographic, KAP and the source of dengue information. We statistically compared the KAP between the two study sites and the pre- and post-test scores to evaluate the health education program. A total of 203 students participated in the survey, and 51.7% of them were flood victims. When comparing the baseline KAP, the respondents from the unflooded area had higher knowledge scores compared to those from the flooded area (*P*<0.05), while non-significant differences were observed in the attitude and practice between the two study areas (*P*>0.05). The health education program significantly improved knowledge and practice in the flooded area and knowledge only in the unflooded area (*P*<0.05). The multinomial regression analysis suggests that age and dengue history are the primary determinants that influence the high practice level in both areas. We suggest the need to increase routine dengue health education programs to all age groups targeting both high and low dengue risk areas, and the necessity to ensure the translation of positive knowledge and attitude changes into real dengue preventive practices.

## Author summary

The KAP research design is widely used in diagnosing the current awareness of any diseases and to evaluate the efficiency of any health-related treatments or intervention programs. Until date, such KAP assessment and health education programs regarding dengue in the disaster-affected region are relatively rare, especially in Malaysia. Providing health education to the community is as important as other vector control strategies such as source reduction and insecticide spraying because none of these will be successful without community involvement is disease prevention. The massive flood that struck the east coast region of Malaysia poses a possible threat in Kelantan for dengue transmission. Dengue KAP assessment associated with health education for the community at risk is essential to find the gaps in the community and also to ensure their understanding in vector biology, disease spread mechanisms and key behaviors that need to be adopted in order to prevent the spread of dengue. The study provides useful content and knowledge that could guide local authorities and health officials to plan and execute health education programs to disaster-affected populations and also unaffected populations to prevent and control dengue.

## Introduction

Continuous heavy monsoon rainfall has bought Malaysia’s east coast to a halt as a massive flood struck the region from mid-December 2014 to January 2015. The most affected states were Kelantan, Terengganu, and Pahang. The National Security Council reported that the 2014 flood was the worst in the history of Kelantan. In the same year, Kelantan faced a devastating period of dengue epidemic with a total of 14,456 cases and 17 deaths [1]. The relationship between flooding and dengue incidence is indirect and complex. Flooding can initially wash away mosquito breeding grounds and cause a reduction in the mosquito population. However, stagnant water remaining after the floodwater has receded could serve as a new and ideal breeding ground for mosquitoes, and thus causes a resurgence in the vector population [2], which might increase the risk for disease transmission.

The State Health Department has carried out routine prevention and control measures such as Thermal Space Spraying, elimination of larval breeding grounds and Ultra Low Volume spraying to prevent the disease spread in the Kelantan State [3]. This was undertaken primarily to avoid a possible dengue outbreak by considering the status of dengue cases in the previous years and the risks posed by the flood in this State [4]. However, it is noteworthy that community awareness and their participation is equally important as other public health interventions to control and prevent dengue spread and infection [5]. Frequent spraying by public health authorities to monitor the vector population is not adequate to control the disease spread. Dengue is a severe yet preventable disease that relies on source reduction and control programs. One of the ways to achieve this is through community awareness and participation in dengue control programs [6,7]. Community involvement in disease prevention and control is attainable through assessment and improvement of people’s knowledge, attitude, and practice (KAP) regarding dengue.

The KAP research design is a widely utilized strategy in diagnosing the current awareness and practices in any diseases and to evaluate the efficiency of any health-related treatments or intervention programs [8]. Providing dengue health education for the community at risk is essential to ensure the understanding of community members in vector biology, disease spread mechanisms and key behaviors that need to be adopted in order to prevent the spread of dengue. Review of literature on dengue health education in dengue-endemic countries has revealed an improvement in knowledge, attitude, and practice. For example, a dengue health education among mothers living in four communes of Southern Vietnam illustrated significant increase in the overall KAP score from pre-KAP to post-KAP [9]. Also, a study conducted among nursing students in Telangana, India regarding the impact on dengue fever (DF) using pre- and post-test design and structured education showed enhancements on the KAP levels [10]. Beckett et al. [11] observed significant improvement in post-test scores after an education program to enhance knowledge and awareness on dengue, conducted among employees of two textile companies in Bandung, Indonesia.

School-based health education is a commendable tool to enhance knowledge and create awareness among school children about the seriousness of dengue since this disease is particularly prevalent among them. In fact, it has been recognized that dengue is most prevalent in the community among the age group of 13 to 35 years old [12], this being the age range including school children as well as young adults. School age children have been encouraged to participate in ongoing household dengue control activities, such as source reduction, as part of dengue control efforts [13]. In Pune, an educational program among high school children significantly increased their knowledge of dengue and its prevention [14]. Similarly, a primary school-based participatory education program in Thailand increased basic knowledge on dengue prevention and control, leading to a reduction in larval indices in the primary school and in the students’ households [15]. To date, such KAP assessment and health education programs regarding dengue in disaster-affected regions have been relatively rare, especially in Malaysia. Most dengue KAP studies in Malaysia have assessed community-based KAP [16,17,18], but without emphasis on dengue health education.

Kelantan State suffered a severe dengue outbreak prior to the region suffering the major flood in December 2014; the State then came to rank third for reported dengue cases in Malaysia. Given the potential for possible dengue outbreak following the flooding in Kelantan, dengue fever can be considered as one of the health priorities in the areas affected by the flood, especially in the ex-dengue hotspot area. Therefore, it is crucial to assess the KAP of the community at risk and to educate them on the seriousness and the prevention of the disease. This study aimed to assess and compare the KAP of school children from the flooded and unflooded area towards DF and to evaluate the effectiveness of dengue awareness health education programs in improving their KAP level.

## Methodology

### Ethics approval and consent to participate

This study has been reviewed and approved by the ethics committee of the School of Biological Sciences, Universiti Sains Malaysia. Permission to conduct the survey was obtained from the respective school administration prior to the survey. Verbal informed consent was obtained from the students’ parents or caretakers by providing them a study information sheet before the survey. Through the sheet, the parents or caretakers were informed about the purpose and content of the study and given the opportunity to agree to or decline their child’s participation in this survey. The verbal agreement was obtained by the students from their parents. The students' assent was obtained by giving a written assent letter attached to the questionnaire before initiating the survey. They were informed that completion of the questionnaire is considered as their assent to take part in the survey. It was emphasized that the participation is voluntary and the confidentially and anonymity of the participants will be retained.

### Study design

A cross-sectional study was conducted to assess and compare the KAP regarding dengue in two sites in Kelantan: Pasir Pekan (flooded area) and Kubang Kerian (unflooded area) by using a pre- and post-test design. A health education program was carried out by distributing a dengue awareness booklet entitled “Dengue and *Aedes* Mosquito: Know Your Enemy” [19] among the students during the interphase of the tests.

### Conceptual framework

**Fig 1:**
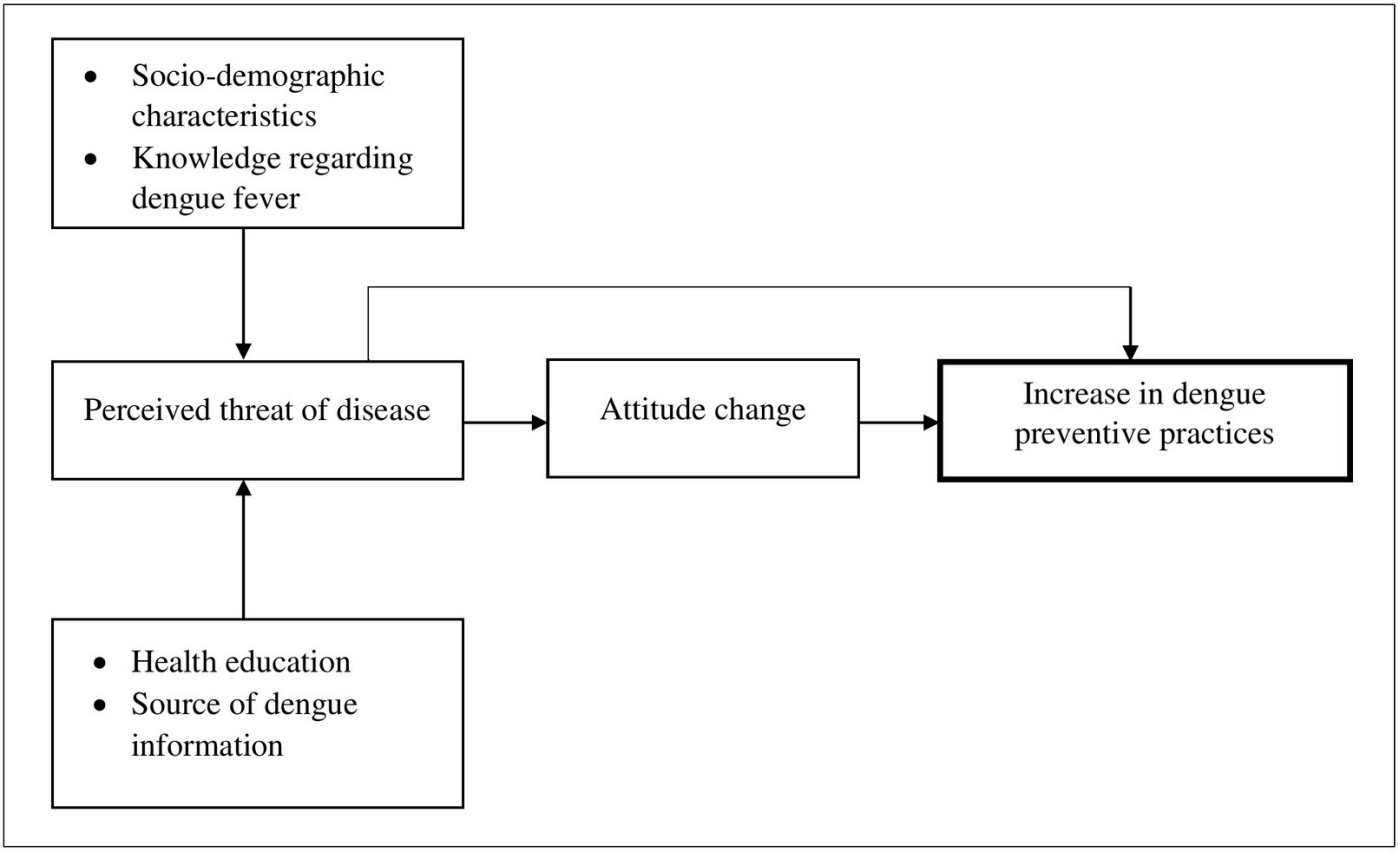
Conceptual framework of the study.

The study was designed and interpreted based on the conceptual framework (Fig 1). The underlying hypothesis is that socio-demographic characteristics and knowledge, along with health education and information regarding dengue, could affect the dengue preventive practices with or without the change in attitude.

### Study site selection

**Fig 2:**
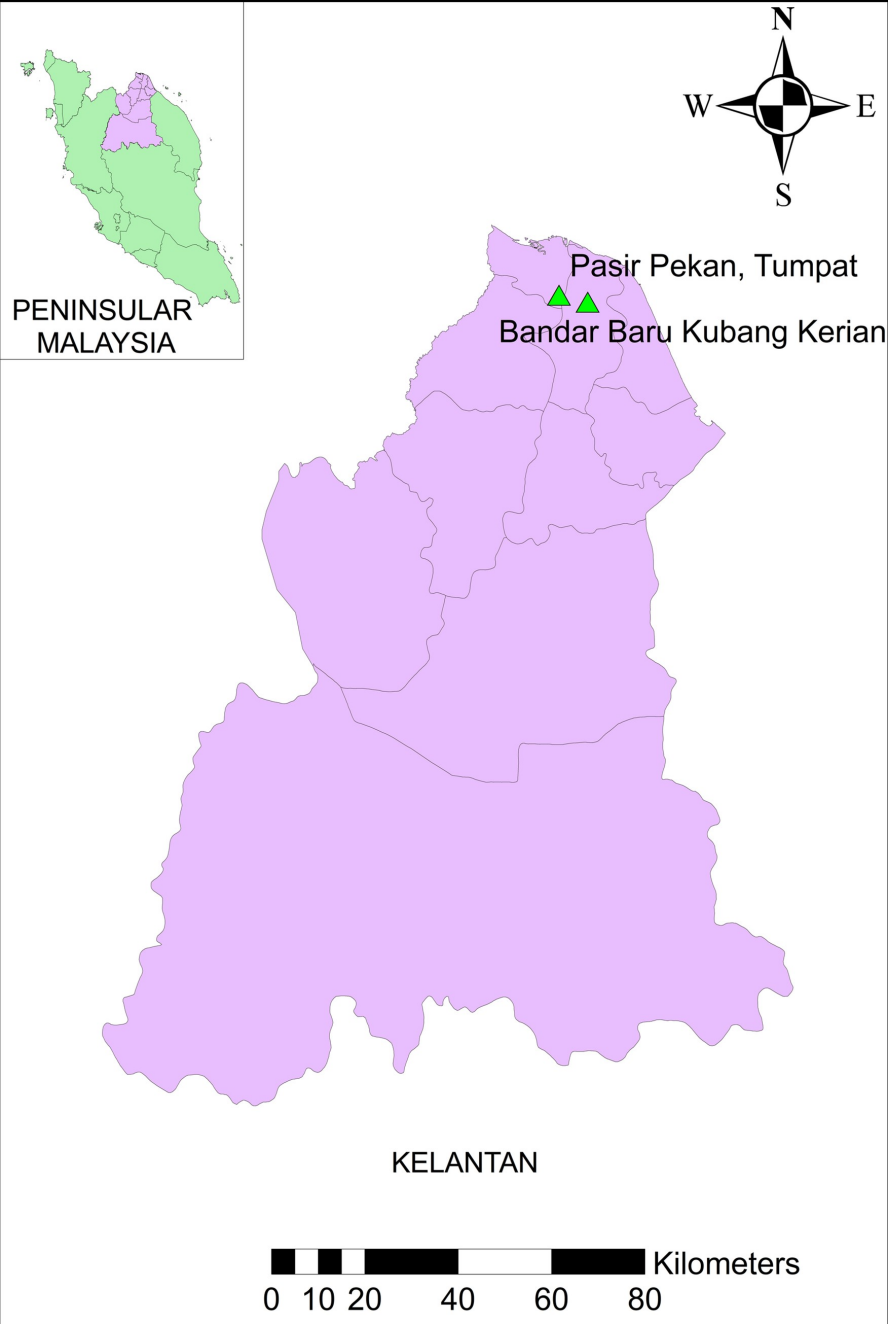
Map of Kelantan, Malaysia, showing two sampling sites of this study, Pasir Pekan, Tumpat, and Bandar Baru Kubang Kerian, Kubang Kerian.

In order to assess and compare the KAP among school children between the flooded and unflooded areas in Kelantan, one school was selected from each region by using convenience sampling. The selected schools in this study were Mahmud Mahyiddin Secondary School, Pasir Pekan, Tumpat (flooded area) and Kubang Kerian Secondary School, Kubang Kerian (unflooded area) (Fig 2). The flooded area is located one km away from the Kelantan River which was the source of the flood in 2014 whereas the unflooded area is located approximately 8 km away from the Kelantan River. Both the selected study sites represent areas with known distribution of *Aedes* mosquitoes, vectors responsible for the spread of dengue. Furthermore, both areas faced a severe dengue outbreak before the flood incidence in December 2014 according to the source provided by Kelantan Health Department.

### Study population

The study was performed among 203 secondary school children between the age of 13–17 years old. The sample was chosen based on the idea explained by Beinner et al. [20] that the youngsters within this age range are able to engage in abstract cognitive thinking and plan theory. This enables them to transform their thinking into systematic practical solutions based on rationale. Another essential point is that at this age level, the perception of environmental awareness extends beyond commonplace limits. In addition, it is expected that teenagers at this age serve as young educators as they tend to share their knowledge and experiences with their family and also to other community members.

### Sample size

The estimation of the minimum required sample size was calculated using Rao Softonline sample size calculator [21]. Since the KAP level of the study population is unknown, we assumed the most statistically conservative response distribution is possible at 50%. To obtain a representative sample of the school children (<900 student population in both schools) with 90% confidence interval and 8% margin of error, we estimated a minimum of 95 students required from each school. A total of 108 students from the flooded and 95 students from the unflooded areas volunteered to participate during the pre-test this study.

### Study instruments

A standardized questionnaire (S1 Appendix) was developed using a baseline from the previous questionnaires on related studies [22–24]. Emphasis was given on the age group that responded to this tool. The questionnaire was translated into Bahasa Malaysia (the national language of Malaysia, S2 Appendix) and was made sure that the original meaning was retained.

Validity is a measure of the extent to which a test measured what it intended to measure [25]. In this study, face validity of the fully developed questionnaire was evaluated by established experts from the Graduate School of Business, Universiti Sains Malaysia to ensure content validity. Amendments were made according to the suggestions. Reliability is a measure of internal consistency of an instrument, ensuring that the test yields the same results in repeated trials using the same data [25]. Before conducting the actual survey, the reliability of the questionnaire was determined by testing it on 30 students and analyzed using Cronbach’s Alpha. The Cronbach’s Alpha coefficient of overall KAP domains was 0.55. While a minimum of 0.80 is considered as acceptable reliability, coefficients in the range of 0.50 to 0.60 which is considered as modest reliability may be acceptable in such cases that the measurement results are used to decide about a group or for research purposes [25].

The dengue awareness booklet was bilingual (English and Bahasa Malaysia). The booklet covers knowledge on DF and *Aedes* mosquitoes, which mainly aimed to expose people to the seriousness of the disease, its spread, the vectors and their characteristics, prevention and control measures to eliminate breeding grounds and prevention of mosquito bites.

### Description of the questionnaire

The questionnaire comprised of 49 questions and statements which were divided into six categories: (1) socio-demographic characteristics (age, gender, family income, type of house, vegetation and mosquito abundance surrounding the house, flood victim and dengue history in the family); (2) knowledge of the vectors, symptoms, transmission and control of dengue; (3) attitude towards dengue prevention; (4) practices regarding dengue prevention (e.g. practices to prevent mosquito breeding and precautions taken to avoid mosquito bites; (5) Practices during flood to prevent dengue infection; (6) Source of information on dengue. The respondents were allowed to choose more than one option for the source of information on dengue.

### Data collection

The questionnaire was handed over to the volunteered students as a pre-assessment (pre-test) prior to the health education program to assess their baseline KAP. They were allocated a reasonable time to finish answering the questionnaire. Overall, it took about 20–30 minutes for them to complete the questionnaire. Shortly after the pre-test, the booklet was distributed to each respondent. The respondents were given a health education lesson which started with a 15 minute lecture using the booklet. In order to ensure the effective dissemination of knowledge among the respondents, interactive sessions such as discussions, briefing and question and answer (Q&A) were included as well. At the end of the session, the respondents were requested to share the messages and booklet they obtained through the study with family and friends. In addition, the respondents were also provided with small gifts with dengue educational messages to further stimulate changes in dengue preventive practices. The same set of questionnaires was re-administered (post-test) to the same group of respondents after a week. The reason for keeping the one-week interval between pre- and post-test was to ensure knowledge retention among the respondents so that the change in the KAP associated with the health education program can be measured. The respondents were again allocated a reasonable amount of time to answer the questionnaire to allow them to recall memory. The questionnaires were not linked individually due to the unequal sample sizes in the pre- and post-test and also to preserve confidentiality. Only those who have participated in the pre-test were allowed to complete the post-test

### Data analysis

The knowledge scores varied from 0–13 points. The original Bloom’s cutoff points [26] were were adapted and modified to classify the scores into three levels: high level (85–100%) with scores between 11–13, moderate level (60–84%) with scores between 8–10 and low level (less than 59%) with scores between 0–7. The attitude section contained 11 positive and negative statements that were rated by using Likert’s scale [27]. For positive statement scores, the rating scale was measured as: strongly agree– 5, agree– 4, neither agree or disagree– 3, disagree– 2 and strongly disagree– 1. For negative statements, the scores were opposite: strongly agree– 1, agree– 2, neither agree or disagree– 3, disagree– 4 and strongly disagree– 5. The individual scores varied from 11 to 55. Responses were summed up, and means were calculated. The scores were categorized into three levels: Positive attitude (44–55), Neutral attitude (38–43) and Negative attitude (11–37) using a modified Bloom’s cut off point. The responses for practice were assessed as zero-one indicator (dummy) variables. The variables were given one for “yes” and zero for “no”. The classification was based on: Good practice (8–9 scores), Fair practice (6–7) and Poor practice (0–5). (modified Bloom’s cut off point,). The section on practices after the flood to avoid dengue infection was assessed by using dummy variables: one for “yes” and zero for “no” based on: Good practice (4–6 scores), Fair practice (2–3) and Poor practice (0–1). (modified Bloom’s cut off point).

The data of the completed questionnaires were entered into Microsoft Excel program and double-checked before analysis. The analysis of the data was performed in the Statistical Package for Social Sciences (SPSS) version 22. Data were checked for normality (Shapiro-Wilk) prior to analysis, and natural log-transformation (ln[y+1]) was made due to the homogeneity of variance. Descriptive statistics in terms of frequency (n), percentage (%), mean and standard deviation (SD) were used to express the data. The socio-demographic variables between the two study areas were compared by using a Chi-square analysis. Fisher’s exact test was used when more than 20% of cells had expected cell counts less than five. For KAP, Independent *t*-test was used to compare pre- and post-tests results and also to compare the baseline information between these two study sites. Even though Independent *t*-test is used to compare means between two independent groups, in order to determine whether there is statistical difference between the groups in this study, it is the only suitable test that can be used to compare the pre- and post-test means. Since the pre- and post-tests were not linked individually, a paired *t*-test could not be performed to analyze the statistical difference between the groups. Correlation and regression analysis were carried out on the pre-test data for explaining the baseline information between the study sites. The correlation coefficient was used to describe the correlation between knowledge-attitude, knowledge-practice, and attitude-practice. Multinomial logistic regression was carried out using JMP statistical package version 13 to identify factors associated with high level of dengue prevention practices. The factors were socio-demographic, knowledge (high, moderate, low) and attitude (positive, neutral, negative) level. A stepwise procedure with a backward method was used to create the best fit model for the factors mentioned above by choosing the most significant values, and it was run separately for flooded and unflooded areas. Least significant factors (*P*>0.05) were excluded from the equation to obtain the best fit reliable equation for the model.

## Results

The total number of students participated in the pre-test was 203 (108 from the flooded area and 95 from the unflooded area) and the post-test was 168 (86 from the flooded area and 82 from the unflooded area).

### Socio-demographic characteristics

Table 1 shows the socio-demographic characteristics of the 203 respondents according to the study site, collected during the pre-test. Of this, 108 (51.7%) were students from the flooded area, and 95 (46.8%) were from the unflooded area. During the post-test, the total number of respondents was 168, of which consist of 86 (51.2%) students from the flooded area and 82 (48.8%) students from the unflooded area. There were about 35 students from both study areas unable to attend the post-test due to exams and school activities. The age of the respondents ranged from 13 to 17 years old; the majority belonged to the age group of 14 years (80: 39.4%). However, not all respondents from all the age groups were able to participate in the survey due to school activities and exams during the survey period. Of the study respondents, 116 (57.1%) were female and 87 (42.9%) were male. The common monthly family income ranged between MYR 900–1500 (MYR: Malaysian Ringgit) (109: 53.7%) in both study sites. Most of the respondents live in Bungalow/Village/Flats type of house (164: 80.8%) and 114 (56.2%) of the respondents stated that their house is surrounded by a moderate level of vegetation. There were about 111 (54.7%) of the total respondents stated that there is a moderate level of mosquito abundance in their neighborhood, while only 10 (4.9%) of the respondents stated severe mosquito abundance with an estimation of more than 100 mosquitoes. Most of the respondents from Mahmud Mahyiddin Secondary School, which we considered as in a flooded area, were flood victims (83: 76.9%) at their residential area, whereas respondents of Kubang Kerian Secondary School, which we considered as in an unflooded area, showed a lesser number of flood victims (22: 23.2%) at their residential area, (*P*<0.05). There were 29 (14.3%) households in total had the history of DF in recent time. The Chi-square test showed that there was no significant difference in fogging frequency between both study sites (*P*>0.05). The majority of the respondents from both areas stated that fogging is carried out at greater than bi-monthly frequency.

**Table 1:** Socio-demographic characteristics.

Characteristics	Flooded area n (%)	Unflooded area n (%)	Total n (%)	*P*-value
**1. Age (years)**				
13	30 (27.8%)	0	30 (14.8%)	
14	33 (30.6%)	47 (49.5%)	80 (39.4%)	
15 16 17	6 (5.6%) 0 39 (36.1%)	0 48 (50.5%) 0	6 (3.0%) 48 (23.6%) 39 (19.2%)	0.000*
**2. Gender**				
Male	47 (43.5%)	40 (42.1%)	87 (42.9%)	0.839
Female	61 (56.5%)	55 (57.9%)	116 (57.1%)	
**3. Average monthly family income**				
More than MYR 3001	12 (11.1%)	26 (27.4%)	38 (18.7%)	
MYR 1501–3000	24 (22.2%)	16 (16.8%)	40 (19.7%)	**0.021**
MYR 900–1500	61 (56.5%)	48 (50.5%)	109 (53.7%)	
Less than MYR 900	11 (10.2%)	5 (5.3%)	16 (7.9%)	
**4. Education level**				
Upper secondary	39 (36.1%)	49 (51.6%)	85 (41.9%)	0.076
Lower secondary	69 (63.9%)	46 (48.4%)	118 (58.1%)	
**5. Type of house**				
Bungalow/Village/Flats	88 (81.5)	76 (80.0)	164 (80.8%)	0.789
Terrace/twin	20 (18.5)	19 (20.0%))	39 (19.2%)	
**6. Was your house affected by flood in Dec 2014?**				
Yes	83 (76.9)	22 (23.2)	105 (51.7%)	**0.000**
No	25 (23.1)	73 (76.8)	98 (48.3%)	
**7. Was your house surrounded by dense vegetation?**				
Many	22 (20.4)	35 (36.8)	57 (28.1%)	**0.015**
Moderate	64 (59.3)	50 (52.6)	114 (56.2%)	
Low or None	22 (20.3)	10 (10.6)	32 (15.8%)	
**8. Mosquito abundance in your neighborhood**				
Severe (>100)	5 (4.6)	5 (5.3)	10 (4.9%)	0.811*
Moderate (50–100)	56 (51.9)	55 (57.9)	111 (54.7%)	
Low (<50)	45 (41.7)	33 (34.7)	78 (38.4%)	
None	2 (1.9)	2 (2.1)	4 (2.0%)	
**9. Have you/your family member been infected by dengue recently?**				
Yes	12 (11.1)	17 (17.9)	29 (14.3%)	0.168
No	96 (88.9)	78 (82.1)	174 (85.7%)	
**10. Fogging frequency in your neighborhood**				
Weekly/Biweekly	4 (3.7)	2 (2.1)	6 (3.0%)	0.943*
Monthly	9 (8.3)	8 (8.4)	17 (8.4%)	
Seldom (>2 months)	81 (75)	73 (76.8)	154 (75.9%)	
Never	14 (13)	12 (12.6)	26 (12.8%)	

Comparison using Chi-square test, significant values are in bold, *P*<0.05. *P*-values with an asterisk (*) are based on Fisher’s exact test.

### Knowledge on dengue

Table 2 shows the distribution of knowledge level on dengue, its spread, the vectors, and the symptoms. Both study sites showed an increase in respondents with “high knowledge” and a decrease in “moderate and low knowledge” from pre- to post-test. The flooded area showed higher increases in the percentage of respondents with “high knowledge” (12.1%) as compared to respondents in the unflooded area (2.3%) (Table 2). There were no respondents reported as “low knowledge” in both study sites in the post-test.

**Table 2:** Distribution of knowledge level regarding dengue, its spread, its vectors, and the symptoms.

	Flooded area			Unflooded area		
Level	Pre-test n (%)	Post-test n (%)	% change	Pre-test n (%)	Post-test n (%)	% change
**High (11–13 scores)**	71 (65.7%)	67 (77.8%)	+12.1	79 (83.1%)	70 (85.4%)	+2.3
**Moderate (8–10 scores)**	36 (33.4%)	19 (22.2%)	-11.2	15 (15.8%)	12 (14.6%)	-1.2
**Low (0–7 scores)**	1 (0.9%)	0 (0%)	-0.9	1 (1.1%)	0 (0%)	-1.1
**Total**	108 (100%)	86 (100%)		95 (100%)	82 (100%)	

The number of respondents scored correctly for each question and the percentage change between pre- and post-test is recorded in Table 3 for both study sites. During both tests, 100% of the respondents agreed that dengue is a serious illness for both study sites. The majority of the respondents (>94%) were able to choose correct answers for questions regarding dengue as a serious illness, dengue symptoms, and prevention of vector breeding ground, including the use of Abate (BASF Agricultural) to kill mosquito larvae. In the case of questions 14, 19, 21, the percentage of respondents answering correctly in the post-tests increased significantly after providing them with a dengue information booklet (*P*<0.05). The questions for which the least number of students scored correctly were questions 19 and 21; this was found for both study sites. For question 19, a significant increase in the percentage of the respondents disagreeing to the statement that mosquitoes prefer to lay eggs in dirty water was observed among the students from the flooded area (*P*<0.05; Table 3) whereas an insignificant increase was observed in the unflooded area (*P*>0.05; Table 3). Similarly. for question 21, a significant increase in the percentage of respondents from the unflooded area disagreeing to the statement that there is proper vaccine or medications available for treatment of dengue was observed (*P*<0.05; Table 3). Whereas, a non-significant increase was observed for the same question in the flooded area (*P*>0.05; Table 3).

**Table 3:** Pre- to post test percentage change in correctly answered questions concerning knowledge, for respondents in flooded and unflooded area.

Questions	Flooded area				Unflooded area			
	Pre-test n (%)	Post-test n (%)	% change	*P*-value	Pre-test n (%)	Post-test n (%)	% change	*P*-value
11. Are you aware of dengue? (yes)	107 (99.1%)	86 (100%)	0.9	0.374	94 (98.9%)	82 (100%)	1.1	0.354
12. Dengue is a virus (yes)	90 (83.3%)	79 (91.9%)	8.6	0.069	92 (96.8%)	80 (97.6%)	-0.8	0.775
13. Dengue is a serious illness (yes)	108 (100%)	86 (100%)	-	-	95 (100%)	82 (100%)	-	-
14. Dengue is transmitted to human by bites of infective mosquitoes (yes)	94 (87.0%)	83 (96.5%)	9.5	**0.014**	88 (92.6%)	80 (97.6%)	5.0	0.125
15. Human infected by dengue by drinking dirty water (no)	100 (92.6%)	77 (89.5%)	-3.1	0.457	88 (92.6%)	76 (92.7%)	0.1	0.990
16. The two main vectors of dengue are *Ae*. *aegypti* and *Ae*. *albopictus* (yes)	97 (89.8%)	81 (94.2%)	4.4	0.260	89 (93.7%)	81 (98.8%)	5.1	0.070
17. Dengue patients will develop symptoms such as severe fever, headache, rashes, deep muscular and joint pain (yes)	104 (96.3%)	83 (96.5%)	0.2	0.937	90 (94.7%)	81 (98.8%)	4.1	0.123
18. Mosquitoes bite only during day (no)	86 (79.6%)	63 (73.3%)	-6.3	0.304	88 (92.6%)	72 (87.8%)	-4.8	0.288
19. The mosquitoes that transmit dengue virus lay their eggs in dirty water (no)	51 (47.2%)	60 (69.8%)	22.6	**<0.001**	43 (45.3%)	46 (56.1%)	10.8	0.152
20. Stagnant water in empty containers, used tires, trash cans and flower pots can be possible breeding sites of mosquitoes (yes)	105 (97.2%)	83 (96.5%)	-0.7	0.778	94 (98.9%)	82 (100%)	1.1	0.354
21. There are proper vaccine/medications available for treatment of dengue (no)	19 (17.6%)	19 (22.1%)	4.5	0.435	22 (23.3%)	43 (42.4%)	29.2	**<0.001**
22. Only way to prevent dengue is by eliminating breeding grounds of dengue (yes)	85 (98.8%)	85 (98.8%)	1.6	0.434	95 (100%)	79 (96.3%)	-3.7	0.083
23. Abate can be used to kill mosquito larvae in water storages (yes)	103(95.4%)	84 (97.7%)	2.3	0.395	89 (93.7%)	80 (97.6%)	3.9	0.204

Comparison using Independent *t*-test, significant values are in bold, *P*<0.05; Correct answers are provided in brackets after each question.

### Attitude towards dengue prevention

In this section, the students answered 11 questions which can give a total score of 55. The distribution of attitude level towards dengue prevention is shown in Table 4. Both study sites showed the same trend, whereby there was an increase in the positive attitude, decrease in the neutral attitude and a slightly or very minimal increase in the negative attitude, which might be due to unequal sample sizes during pre- and post-tests. The unflooded area showed higher increases in respondents with a “positive attitude” (10.4%) as compared to respondents in the flooded area (5.8%) (Table 4).

**Table 4:** Distribution of attitude levels toward dengue prevention.

	Flooded area			Unflooded area		
Level	Pre-test n (%)	Post-test n (%)	% change	Pre-test n (%)	Post-test n (%)	% change
**Positive (44–55 scores)**	19 (17.6%)	20 (23.4%)	+5.8	11 (11.6%)	18 (22.0%)	+10.4
**Neutral (33–43 scores)**	87 (80.6%)	63 (73.3%)	-7.3	76 (80.0%)	57 (69.5%)	-10.5
**Negative (0–32 scores)**	2 (1.9%)	3 (3.5%)	+1.6	8 (8.4%)	7 (8.5%)	+0.1
**Total**	108 (100%)	86 (100%)		95 (100%)	82 (100%)	

Table 5 summarizes the frequency and percentage for each question of the attitude towards dengue prevention in both study sites. Respondents from both study sites showed significant changes in scores for the statement that dengue patients need immediate treatment and hospitalization (*P*<0.05). The respondents responded variously to the first statement in this section that they are at risk of getting dengue fever. In both study sites, there were only less than 26% of the respondents (agreed and strongly agreed) thought that they were at the risk of infected with dengue during the pre-test and the percentage insignificantly increased to 37% in the post-test. Most of the respondents strongly agreed or agreed that they play a vital role in curbing DF in their surroundings. The respondents from both the study sites also seem to believe that a healthy person will not get dengue infection as most of them strongly agreed or agreed with this statement.

**Table 5:** Frequency and percentage of the respondents by the attitude towards dengue prevention for each individual item during pre- and post-test in the flooded and unflooded area.

	Flooded area				Unflooded area			
Item	Pre-test	Post-test	% change	*P*-value	Pre-test	Post-test	% change	*P*-value
Positive statements								
24. You are at risk of getting dengue fever.								
**Strongly Disagree**	13 (12.0%)	5 (5.8%)	-6.2	0.345	11 (11.6%)	7 (8.5%)	-3.1	0.086
**Disagree**	30 (27.8%)	30 (34.9%)	7.1		21 (22.1%)	15 (18.3%)	-3.8	
**Neither Agree or Disagree**	37 (34.3%)	23 (26.7%)	-7.6		42 (44.2%)	30 (36.6%)	-7.6	
**Agree**	21 (19.4%)	21 (24.4%)	5.0		15 (15.8%)	19 (23.2%)	7.4	
**Strongly Agree**	7 (6.5%)	7 (8.1%)	1.6		6 (6.3%)	11 (13.4%)	7.1	
26.Dengue patient needs immediate treatment and hospitalization.								
**Strongly Disagree**	3 (2.8%)	1 (1.2%)	-1.6	**<0.001**	0	1 (1.2%)	1.2	**<0.001**
**Disagree**	0	1 (1.2%)	1.2		2 (2.1%)	3 (3.7%)	1.6	
**Neither Agree or Disagree**	3 (2.8)	2 (2.3%)	-0.5		3 (3.2%)	6 (7.3%)	4.1	
**Agree**	24 (22.2%)	26 (30.2%)	8.0		25 (26.3%)	33 (40.2%)	13.9	
**Strongly Agree**	78 (72.2%)	56 (65.1%)	-7.1		65 (68.4%)	39 (47.6%)	-20.8	
32. Sleeping in mosquito/bed net will prevent mosquito bites and dengue infections.								
**Strongly Disagree**	2 (1.9%)	0	-1.9	0.907	1 (1.1%)	1 (1.2%)	-0.1	**0.009**
**Disagree**	9 (8.3%)	2 (2.3%)	5.0		5 (5.3%)	5 (6.1%)	0.8	
**Neither Agree or Disagree**	17 (15.7%)	12 (14.0%)	-1.7		12 (12.6%)	15 (18.3%)	5.7	
**Agree**	52 (48.1%)	48 (55.8%)	7.7		50 (52.6%)	42 (51.2%)	-1.4	
**Strongly Agree**	28 (25.9%)	24 (27.9%)	2.0		27 (28.4%)	19 (23.2%)	-5.2	
33. You will allow health inspectors to conduct inspections for larval breeding sources inside/outside the house.								
**Strongly Disagree**	0	1 (1.2%)	1.2	**0.008**	0	3 (3.7%)	-3.7	0.459
**Disagree**	1(0.9%)	0	-0.9		1 (1.1%)	1 (1.2%)	0.1	
**Neither Agree or Disagree**	13 (12.0%)	7 (8.1%)	-3.9		12 (12.6%)	10 (12.2%)	-0.4	
**Agree**	40 (37.0%)	41 (47.7%)	10.7		42 (44.2%)	32 (39.0%)	-5.2	
**Strongly Agree**	54 (50.0%)	37 (43.0%)	-7.0		40 (42.1%)	36 (43.9%)	1.8	
34. You play a vital role to curb dengue fever in your surroundings.								
**Strongly Disagree**	2 (1.9%)	1 (1.2%)	-0.7	0.072	2 (2.1%)	3 (3.7%)	1.6	0.076
**Disagree**	5 (4.6%)	0	-4.6		4 (4.2%)	0 (0.0%)	-4.2	
**Neither Agree or Disagree**	9 (8.3%)	7 (8.1%)	-0.2		11 (11.6%)	12 (14.6%)	3.0	
**Agree**	46 (42.6%)	36 (41.9%)	-0.7		37 (38.8%)	39 (47.6%)	8.8	
**Strongly Agree**	46 (42.6%)	42 (48.8%)	6.2		41 (43.2%)	28 (34.1%)	-9.1	
Negative statements								
25. Dengue fever can be cured.								
**Strongly Disagree**	17 (15.7%)	6 (7.0%)	-8.7	0.290	16 (16.8%)	7 (8.5%)	-8.3	0.867
**Disagree**	52 (48.1%)	27 (31.4%)	-16.7		40 (50.5%)	33 (40.2%)	-10.3	
**Neither Agree or Disagree**	17 (15.7%)	15 (17.4%)	1.7		28 (29.5%)	26 (31.7%)	2.2	
**Agree**	17 (15.7%)	25 (29.1%)	13.4		3 (3.2%)	11 (13.4%)	10.2	
**Strongly Agree**	5 (4.6%)	13 (15.1%)	10.5		0	5 (6.1%)	6.1	
27. It is possible to recover from dengue fever by eating paracetamol.								
**Strongly Disagree**	4 (3.7%)	3 (3.5%)	-0.2	0.187	5 (5.3%)	4 (4.9%)	-0.4	0.212
**Disagree**	14 (13.0%)	30 (34.9%)	21.9		12 (12.6%)	20 (24.4%)	11.8	
**Neither Agree or Disagree**	56 (51.9%)	34 (39.5%)	-12.4		43 (45.3%)	28 (34.1%)	-11.2	
**Agree**	26 (24.1%)	16 (18.6%)	-5.5		27 (28.4%)	22 (26.8%)	-1.6	
**Strongly Agree**	8 (7.4%)	3 (3.5%)	-3.9		8 (8.4%)	8 (9.8%)	1.4	
28. Eradication of mosquito breeding ground is the responsibility of public health authorities and volunteers.								
**Strongly Disagree**	31 (28.7%)	14 (16.3%)	-12.4	0.579	29 (30.5%)	10 (12.2%)	-18.3	0.089
**Disagree**	24 (22.2%)	21 (24.4%)	2.2		15 (15.8%)	18 (22.0%)	6.2	
**Neither Agree or Disagree**	14 (13.0%)	12 (14.4%)	1.4		14 (14.7%)	21 (25.6%)	10.9	
**Agree**	23 (21.3%)	25 (29.1%)	7.8		21 (22.1%)	24 (29.3%)	7.2	
**Strongly Agree**	16 (14.8%)	14 (16.3%)	1.5		16 (16.8%)	9 (11.0%)	-5.8	
29. Elimination of larval breeding ground should be conducted every 1–2 times/year.								
**Strongly Disagree**	10 (9.3%)	4 (4.7%)	-4.6	**0.044**	10 (10.5%)	6 (7.3%)	-3.2	0.355
**Disagree**	8 (7.4%)	14 (16.3%)	8.9		15 (15.8%)	19 (23.2%)	7.4	
**Neither Agree or Disagree**	8 (7.4%)	14 (16.3%)	8.9		13 (13.7%)	12 (14.6%)	0.9	
**Agree**	42 (38.9%)	34 (39.5%)	0.6		36 (37.9%)	26 (31.7%)	-6.2	
**Strongly Agree**	40 (37.0%)	20 (23.3%)	-13.7		21 (22.1%)	19 (23.2%)	1.1	
30. Only fogging is enough to control the mosquito population.								
**Strongly Disagree**	2 (1.9%)	5 (5.8%)	3.9	0.654	3 (3.2%)	2 (2.4%)	-0.8	0.300
**Disagree**	18 (16.7%)	15 (17.4%)	0.7		24 (25.3%)	10 (12.2%)	-13.1	
**Neither Agree or Disagree**	29 (26.9%)	28 (32.6%)	5.7		25 (26.3%)	29 (35.4%)	9.1	
**Agree**	52 (48.1%)	30 (34.9%)	-13.2		28 (29.5%)	30 (36.6%)	7.1	
**Strongly Agree**	7 (6.5%)	8 (9.3%)	2.8		15 (15.8%)	11 (13.4%)	-2.4	
31. A healthy person will not get dengue infection.								
**Strongly Disagree**	4 (3.7%)	3 (3.5%)	-0.2	0.137	0	3 (3.7%)	3.7	0.635
**Disagree**	5 (4.6%)	7 (8.1%)	3.5		6 (6.3%)	7 (8.5%)	2.2	
**Neither Agree or Disagree**	18 (16.7%)	13 (15.1%)	-1.6		15 (15.8%)	14 (17.1%)	1.3	
**Agree**	45 (41.7%)	38 (44.2%)	2.5		48 (50.5%)	40 (48.8%)	-1.7	
**Strongly Agree**	36 (33.3%)	25 (29.1%)	-4.2		26 (27.4%)	18 (22.0%)	-5.4	

Comparison using Independent *t*-test, significant values are in bold, *P*<0.05

### Practices regarding dengue prevention

Table 6 shows that the majority of the respondents from both the study sites showed an increase in good practice level from pre- to post-test especially those from the flooded area (17.1%). Respondents from the flooded area showed a more significant increase in practice from pre- to post-test for question no. 3, 5 and 6, whereas respondents from the unflooded area only showed a significant increase in question 9 (*P*<0.05) (Table 7). However, it can be concluded that most of the respondents already have a good practice level, even at the pre-test level with their scores for each question were mostly more than 90%, except for question 6, which states that wearing light-colored clothes to prevent mosquito bites. Thus, it indicates that the respondents were less aware of this preventive measure and the health education booklet has helped them to improve their practice level towards dengue prevention by 21%.

**Table 6:** Distribution of practice level in dengue prevention.

	Flooded area			Unflooded area		
Level	Pre-test n (%)	Post-test n (%)	% change	Pre-test n (%)	Post-test n (%)	% change
**Good (8–9 scores)**	82 (75.9%)	80 (93.0%)	+17.1	85 (89.5%)	76 (92.7%)	+3.2
**Fair (6–7 scores)**	24 (22.2%)	5 (5.9%)	-16.3	9 (9.5%)	6 (7.3%)	-2.2
**Poor (0–5 scores)**	2 (1.9%)	1 (1.2%)	-0.7	1 (1.1%)	0 (0%)	-1.1
**Total**	108 (100%)	86 (100%)		95 (100%)	82 (100%)	

**Table 7:** Frequency and percentages of the questions on the practice of dengue prevention in the flooded and unflooded area.

	Flooded area				Unflooded area			
Items	Pre-test	Post-test	% change	*P*-value	Pre-test	Post-test	% change	*P*-value
	n (%)	n (%)			n (%)	n (%)		
35. Eliminate standing water around the house to eliminate mosquitoes	108 (100%)	86 (100%)	-	-	94 (98.9%)	82 (100%)	1.1	0.354
36. Rear mosquito eating fish in tanks/pools to reduce mosquitoes	98 (90.7%)	83 (96.5%)	5.8	0.095	92 (96.8%)	77 (93.9%)	-2.9	0.351
37. Turn over/Cover tightly containers to avoid water collection	102 (94.4%)	86 (100%)	5.6	**0.014**	94 (98.9%)	80 (97.6%)	-1.3	0.479
38. Use mosquito bed nets to avoid mosquito bites	104 (96.3%)	82 (95.3%)	-1.0	0.743	88 (92.6%)	79 (96.3%)	3.7	0.278
39. Use insecticide to kill mosquitoes	89 (82.4%)	83 (96.5%)	14.1	**<0.001**	91 (95.8%)	78 (95.1%)	-0.7	0.832
40. Wear light colored and fully covered clothes to avoid mosquito bite	74 (68.5%)	77 (89.5%)	21.0	**<0.001**	64 (67.4%)	65 (79.3%)	11.9	0.073
41. Clear up bushes/vegetation around house to reduce mosquitoes	100 (92.6%)	84 (97.7%)	5.1	0.094	87 (91.6%)	75 (91.5%)	-0.1	0.978
42. Cleaning of garbage/trash around house	107 (99.1%)	86 (100%)	0.9	0.374	94 (98.9%)	81 (98.8%)	-0.1	0.917
43. Government spray insecticide (fogging) to kill mosquitoes	104 (96.3%)	79 (91.9%)	-4.4	0.205	95 (100%)	78 (95.1%)	-4.9	**0.045**

Comparison using Independent *t*-test, significant values are in bold, *P*<0.05

### Practices during the flood to avoid dengue infection

The practices followed by the respondents during the flood to prevent the dengue infection are summarized in Table 8. In most of the statements, more than 90% of the respondents agreed that they followed good preventions to avoid mosquito bites by sleeping in mosquito impregnated net, spraying aerosol and the use of mosquito coils. There were only about 7.4% and 2.1% of the respondents from the flooded and unflooded areas stated that they did not take any precautions.

**Table 8:** Frequency and percentages of practices during the flood to avoid dengue infection.

Items	Flooded	Unflooded	*P*-value
	area, n (%)	area, n (%)	
44. Sleep inside mosquito impregnated nets	96 (88.9%)	87 (91.6%)	0.523
45. Use smoke/mosquito coil to drive away mosquitoes	99 (91.7%)	86 (90.5%)	0.777
46. Use insecticide spray/aerosol to kill mosquitoes	101 (93.5%)	92 (96.8%)	0.267
47. Stay indoor	91 (84.3%)	80 (84.2%)	0.992
48. Did not take any precautions	8 (7.4%)	2 (2.1%)	0.072

Comparison using Independent *t*-test, significant values are in bold, *P*<0.05

### Source of information on dengue

**Fig 3:**
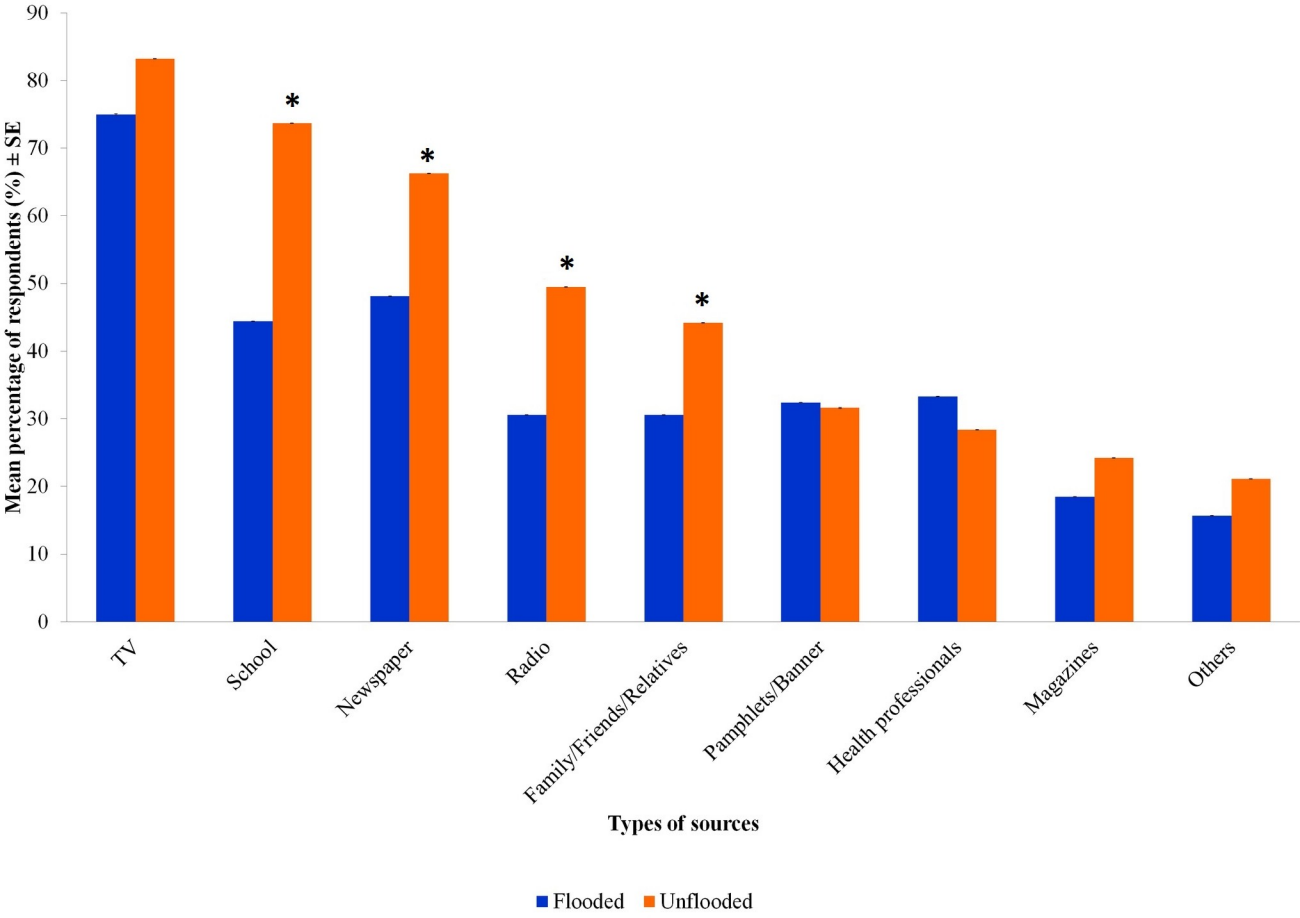
Graph showing the respondents' source of information on the dengue in both study sites. Comparison using Independent *t*-test, values followed by identical letter do not differ significantly, *P*<0.05 between flooded and unflooded areas.

The source of information on dengue among the respondents is displayed in Fig 3. In this part, participants were allowed to select more than one source of information on the dengue. Most of the respondents received information through television followed by newspapers and schools. However, the sources of information from the school, newspaper, radio and from human sources received by the respondents from the unflooded area were significantly higher than those in the flooded area (*t*-test; *P*<0.05) (Fig 3). The least information was gathered from pamphlet/banner and magazines (Fig 3).

### Overall comparisons between variables

Overall, all of the criteria tested showed a numerical increase between pre- and post-test for both study sites. However, the significant increase was only observed in certain criteria only: School in the flooded area showed a significant increase in mean knowledge and practice (*P*<0.05) whereas school in the unflooded area showed a significant increase in mean knowledge only (*P*<0.05) (Table 9). The comparison of KAP means scores using Independent *t*-test between flooded and unflooded areas revealed that students from the unflooded area had relatively higher levels of knowledge (*P*<0.05) as compared to students from the flooded area (Table 9). Insignificant differences were observed for attitude and practice criteria between these two areas.

**Table 9:** Comparison of KAP mean scores between pre- and post-tests and flooded and unflooded areas.

	Comparison of KAP scores						
	Tests						Areas
Criteria	Flooded Mean±SD		*P*- value	Unflooded Mean±SD		*P*- value	*P*- value
	Pre-test (n = 108)	Post-test (n = 86)		Pre-test (n = 95)	Post-test (n = 82)		
**Knowledge**	10.82±1.14	11.26±1.11	**0.009**	11.23±1.12	11.74±1.12	**0.004**	**0.015**
**Attitude**	39.44±3.89	40.03±4.37	0.322	38.84±4.27	39.09±4.84	0.613	0.253
**Practice**	8.20±0.97	8.67±0.73	**<0.001**	8.41±0.83	8.48±0.77	0.589	0.111

Comparison using Independent *t*-test, significant values are in bold, *P*<0.05

### Correlation between knowledge, attitude and practice scores

The correlation between KAP scores revealed a significant positive correlation between knowledge-attitude for the flooded area and knowledge-practice for the unflooded area (*P*<0.05). No significant correlation was observed between attitude and practice in both locations. The degree of correlation was found to be low (r_s_<0.5) for both locations (Table 10).

**Table 10:** Correlation between knowledge, attitude and practice scores for the flooded and unflooded area.

Variables	Flooded area Unflooded area			
	r_s_	*P*-value	r_s_	*P*-value
Knowledge-Attitude	0.222	**0.021**	0.062	0.552
Knowledge-Practice	0.154	0.112	0.231	**0.024**
Attitude-Practice	-0.128	0.187	0.099	0.341

r_s_: Spearman rank correlation coefficients, significant values are in bold, *P*<0.05.

### Association of socio-economic factor, knowledge and attitude scores on practice level

Tables 11 and 12 shows multinomial regression analysis of the associations between socio-demographic variables, knowledge and attitude levels on the practice (based on our conceptual framework in Fig 1). The presence of mosquitoes (mosquito abundance) in neighborhoods was more likely to have high dengue preventive practices in the flooded area (*P*<0.05). Respondents with the high and moderate level of knowledge and attitude were more likely to have high practice levels in the flooded area (*P*<0.05). In both locations, age and dengue history were common factors that influence the high dengue preventive practice levels (*P*<0.05). Gender, income, house type, and flood victims were not significantly associated with higher level of dengue prevention practice in the flooded area, whereas, in the unflooded area, the non-significant associates were flooded victim, vegetation and attitude level (*P*>0.05). A best-fit model was created by using the most significant factors as predictors for high practice level (Table 13).

**Table 11:** Multinomial regression analysis for flooded area.

Source	L-R ChiSquare	df	*P*-value
**Age (AGE)**	31.12	2	**<0.0001**
**Gender (G)**	0.03	2	0.9865
**Income (I)**	11.23	5	0.0816
**House type (HT)**	0.09	2	0.9570
**Flood victim (FV)**	1.26	2	0.5328
**Mosquito abundance (MA)**	41.19	6	**<0.0001**
**Dengue history (DH)**	37.15	2	**<0.0001**
**Knowledge (K)**	18.84	4	**0.0008**
**Attitude (A)**	10.47	4	**0.0332**

Multinomial regression JMP (Poor practice as reference category), significant values are in bold, *P*<0.05, df: degree of freedom, L-R: Likelihood-ratio

**Table 12:** Multinomial regression analysis for the unflooded area.

Source	L-R ChiSquare	df	*P*-value
**Age (AGE)**	8.97	2	**0.0034**
**Flood victim (FV)**	15.27	2	0.0622
**Vegetation (VG)**	5.50	6	0.4380
**Dengue history (DH)**	5.05	2	**0.0332**
**Attitude (A)**	1.06	4	0.4961

Multinomial regression JMP (Poor practice as reference category), significant values are in bold, *P*<0.05.

**Table 13:** Multinomial regression showing the best fit model for predictors of practice level.

Area	Practice level	Model
Flooded area (R^2^ value = 0.3263)	Practice (High/Poor)	y = -231.871 int + 15.080 AGE—28.240 MA 1 + 219.474 MA3–36.475 MA4 + 36.416 DH2 + 33.341 K1–125.804 K2–59.361 A1–5.275 A2
Unflooded area (R^2^ value = 0.3577)	Practice (High/Poor)	y = -263.559 int + 19.275 AGE + 19.061 DH2

Multinomial regression JMP (Poor practice as reference category), int: Intercept; MA: Mosquito abundance; DH: Dengue History; K: Knowledge; A: Attitude; *P*-value is based on 2 log-likelihood regression using backward stepwise method, significant values are in bold, *P*<0.05.

## Discussion

The findings of this study should be interpreted by considering carefully the limitations. The first limitation is in terms of the proportion of the participants in both study sites, The number of participants from both schools was unequal as the participation of the students was voluntary. The ratio between the two schools was 1.14, which means that there were 114 students (flooded) to every 100 students in another school (unflooded). Besides, the number of participants during pre- and post-tests from the same school was unequal due to several factors such as the absence of students, the involvement of students in the other school activities and exams. Thus, a statistical comparison was made on overall mean changes instead of individual score changes. Another limitation was the survey questionnaires. We had to adjust the questionnaire content according to the level that is suitable for the understanding of the students. Some criteria had to be excluded from the survey leaving many aspects of the knowledge remaining unexplored.

Although flood is an annual occurrence in most of the states in Malaysia, including Kelantan, KAP studies involving dengue, and its prevention among people, are still lacking in the disaster-affected region. As to our knowledge, only one baseline study was conducted to assess the KAP among the community in Kampung Bayam, Kubang Kerian, Kelantan by Rahman & Zamri [28]. Assessing people’s KAP while educating them on the seriousness and the prevention of the disease after a disaster-affected the region is important to prevent the disease outbreak.

When comparing the knowledge level in the pre-test, the students from the unflooded area had significantly higher knowledge than the students from the flooded area, and we can relate this to a higher number of dengue cases in the family histories of the former. This finding is in line with Wong et al. [23] where survey participants who previously experienced dengue had significantly higher knowledge scores as compared to those who never experienced it. This is also might be due to the frequent dengue outbreak in the unflooded area, as reported by the local health authorities. Thus, awareness campaigns were carried out more frequently in the outbreak region than to those living in the non-outbreak region. This finding also could be attributed to the locations of the schools. The school in the unflooded area is located close to the public university and general hospital, which makes it possible for the students to be exposed to various health education programs and thus, to have better knowledge about dengue and the vectors. Despite the significant difference in knowledge level, we did not find any statistically significant differences in attitude and practice level between the two schools. Since both the locations were ex-dengue hotspots, we could expect almost the same level of practice among the respondents.

The study showed a significant increase in knowledge scores from pre- to post-test in both study sites. This was well observed especially for the questions that tested the knowledge regarding mosquito egg-laying preferences and the availability of dengue vaccine. The questionnaires revealed that the respondents had the misconception that mosquitoes preferentially lay eggs in dirty water and that prior to the dengue health awareness program, they believed that there is an effective vaccine or medications available to treat dengue. However, an increase in the percentage of the respondents disagreeing with those statements was observed, which suggested that providing the booklet led the respondents to realize that dengue vectors prefer to lay eggs in clear water, and to become aware of the lack of an effective dengue vaccine. This shows that the booklet was able to increase their knowledge on dengue, its vectors, and other important information through the health education program. The method of the survey conducted can be viewed as an educational method. The briefing, discussion, and Q&A session were also conducted during delivery of the booklet. The study is in agreement with other health educational studies that knowledge increased when the study population was supplied with health educational materials [13, 23, 29, 30].

The respondents did not show a significant increase in attitude scores after health education. A similar observation was obtained by Lennon and Coombs [31] when assessing attitudes-beliefs change after conducting dengue educational board game. They rationalized their finding with limitations in their study that it only involves one treatment, which is the board game that they used as a tool for dengue hemorrhagic fever health education. Changing attitudes and beliefs related to health requires multiple treatments, processes and a longer period [32]. Since our study also involves only one-time health education, it is quite difficult to expect sudden changes in attitudes in a very short period.

Regarding the dengue prevention practices, the mean scores increased in both study sites but a significant increase was observed in the flooded area. More than 90% of the respondents stated that they performed practices such as rearing mosquito larvivorous fish in tanks/pools, turning over containers to avoid water collection, clearing up bushes/vegetation and garbage around the house to eliminate mosquito breeding sites and the use of mosquito bed nets to avoid mosquito bites. One of the reasons for the high score in practice compared to knowledge and attitude in this study is probably due to items listed under practice section were related to their routine activities in their house or school for controlling mosquito breeding grounds. Whereas, the content for knowledge and attitude sections were more specific and detailed on dengue and its vector. However, we could not determine in our study how all of these selected practices are actually translated into practices in reality. Based on the answers given, fewer respondents practiced wearing light-colored clothes to avoid mosquito bites. This indicates that they were less aware of the information that mosquitoes are attracted to dark colors compared to the bright colors [33]. A similar finding was reported among the Malaysian public by Wong et al. [23], which only one-third of the study participants reported that they wear bright-colored clothing to avoid mosquito bites.

In this study, television was the main source of information among the students from both study areas. Similar results were reported by several other studies whereby television was found as the main source of information [10, 17, 24, 34]. This was followed by school, newspaper, and radio as a source of information on dengue. Since local television channels disseminate various information regarding dengue mostly in the national language of Malaysia, this might be the reason television was chosen by the majority. There was only 30% of the respondents received information regarding dengue from health professionals. This indicates that there is still a lack of dengue health educations by professionals in the study area, apart from programs conducted at the school level to create awareness for dengue prevention.

In this study, we found a significant correlation between knowledge-practice in the unflooded area and knowledge-attitude in the flooded area, but no correlation was observed for the attitude-practice in both areas. Our finding is in line with several other KAP studies [7, 16, 22, 35] which reported a correlation between knowledge and practice, but in contrary with other KAP studies in India [10], Jamaica [36] and the Philippines [8] where an insignificant correlation between knowledge and practice was observed. Knowledge plays an important role in creating awareness and thus in determining community participation in vector control activities [37]. This suggests that more emphasis should be put on dengue educational programs to increase people’s knowledge about dengue, especially on the severity of the disease and preventive measures. However, our study demonstrated a weak correlation between knowledge, attitude, and practice (r_s_<0.5). In a similar study conducted in Nepal, a weak correlation between KAP variables was observed [22]. Based on these findings, one could propose that knowledge does not always translate into attitude change or causes an increase in practice level. Though it was not our goal to investigate how the perceived knowledge has been translated into practices in real life, investigating such gaps could provide a better understanding peoples’ awareness level.

The finding from the multinomial regression suggests that age and dengue history are the main determinants that influence the high practice level. In the flooded area, the dengue preventive practices were influenced by age, mosquito abundance, dengue history, knowledge and attitude level whereas in the unflooded area, the practice level was influenced only by age and dengue history. It is important to note that age plays a significant role in high dengue preventive practices; thus, health education should focus on all age groups to create awareness at a younger age. However, it is also equally important to ensure that the dengue preventive practices are carried out under the supervision of the parents, guardians or teachers if it involves younger age group (<18 years old). Since both of the study sites were spotted as dengue hotspots, most of the respondents had previously been exposed to dengue. Thus, we could assume that knowledge transfer happens within the family and the dengue preventive practices also improve in such families. We also found that mosquito abundance affects the practice level in the flooded area. The presence of a large number of mosquitoes not only causes a nuisance to people but also increases the chances of virus-carrying mosquitoes which in turn increases the risk of dengue spread among them. This finding could be explained by the constructs in Health Belief Model (HBM) whereby high perceived susceptibility to dengue infection leads to higher dengue preventive practices. Perhaps, the combination of increased mosquito bites and dengue history or current dengue outbreak causes the people to be more concerned about these and they are more likely to practice dengue preventive practices. Similarly, Wong et al. [23] reported that neighborhood with a low density of mosquitoes was less likely to practice dengue prevention compared to the neighborhood with a high density of mosquito. Concerning this, dengue health education should also be targeted toward areas with low mosquito abundance to improve their preventive practices on dengue. People tend to have a misconception that if the mosquito prevalence is low, they can neglect the dengue preventive practices and other precautionary measures against dengue.

In this study, Cronbach’s Alpha coefficient of overall KAP domains was 0.55, which indicates modest reliability. Hence, the modest level is acceptable, and it seems no issue of lack of validity of the questionnaires used in the present study. In addition, only those participants who took part in the pre-test were allowed to complete the post-test, thus the pre- and post-tests were not individually linked, and socio-demographic information was collected only during the pre-test. Thus, there might be a possibility that those who have completed the pre-test might have differed from those completed the post-test. Despite all that, our assessment and educational program were considered successful in assessing the KAP and to increase the knowledge in dengue among the study participants over a short period.

## Conclusions

It is essential for the community and the public health officials to understand the disease transmission risk following flooding. Assessment of the KAP level of the school children revealed a high level of knowledge on dengue, neutral attitude and a good level of practice. The dengue awareness health education program successfully improved students’ knowledge related to dengue in both schools. It is important to educate children from the root so that individuals with good KAP can be created. Such education programs should also be directed towards elder populations in the future to study their KAP related to dengue. The findings suggest the need to target dengue education programs to communities with lower dengue risks and mosquito densities where the public perceives a lower chance of dengue infection. However, the efforts to curb dengue spread in high-risk areas should always be given priority. The study also suggests further emphasis of people’s knowledge and attitude and the need for approaches to ensure the actual translation of positive knowledge and attitude changes into real dengue preventive practices. The study provides useful content and knowledge that could guide local authorities and health officials to plan and execute health education programs to disaster-affected populations and also unaffected populations to prevent and control dengue.

## Supporting information

S1 Checklist STROBE Checklist (DOCX)

S1 Appendix Questionnaire in English. (DOCX)

S2 Appendix Questionnaire in Bahasa Malaysia. (DOCX)
