# Supplementary material for: School-based health education for dengue control in Kelantan, Malaysia: Impact on knowledge, attitude and practice
Source: PLoS Negl Trop Dis. 2020 Mar 27;14(3):e0008075. doi: 10.1371/journal.pntd.0008075 (PMC7141698; doi:10.1371/journal.pntd.0008075)
Supplement: S1 Checklist — (DOCX) [file pntd.0008075.s001.docx]

STROBE Statement—checklist of items that should be included in reports of observational studies

|  | Item No. | Recommendation | Page  No. | Relevant text from manuscript |
| --- | --- | --- | --- | --- |
| **Title and abstract** | 1 | (*a*) Indicate the study’s design with a commonly used term in the title or the abstract | 1 | School-based health education for dengue control in Kelantan, Malaysia: Impact on Knowledge, Attitude and Practice |
|  |  | (*b*) Provide in the abstract an informative and balanced summary of what was done and what was found | 2 | This study assessed the KAP regarding dengue among schoolchildren from flooded and unflooded areas and evaluated the effectiveness of the dengue health education program in improving their KAP level.The health education program significantly improved the knowledge and practice (*P*<0.05). The multinomial regression analysis suggested that age and dengue history are the primary determinants that influence the high practice level in both areas. |
| Introduction | | | |  |
| Background/rationale | 2 | Explain the scientific background and rationale for the investigation being reported | 3-5 | Until date, KAP assessment and health education programs regarding dengue in the disaster-affected region are relatively rare, especially in Malaysia. Providing dengue health education for the community at risk is essential to ensure the understanding of community members in vector biology, disease spread mechanisms and key behaviors that need to be adopted in order to prevent the spread of dengue. |
| Objectives | 3 | State specific objectives, including any prespecified hypotheses | 5 | This study aims to assess the KAP of schoolchildren from the flooded and unflooded area towards DF and to evaluate the effectiveness of dengue awareness health education program in improving their KAP level. |
| Methods | | | |  |
| Study design | 4 | Present key elements of study design early in the paper | 6 | A descriptive cross-sectional study was conducted to assess the KAP regarding dengue in two sites in Kelantan: Pasir Pekan (flooded area) and Kubang Kerian (unflooded area) by using a pre- and post-test design. A health education program was carried out by distributing a dengue awareness booklet entitled “Dengue and *Aedes* Mosquito: Know Your Enemy” during the interphase of the tests. The booklets were distributed among the students after the pre-test. |
| Setting | 5 | Describe the setting, locations, and relevant dates, including periods of recruitment, exposure, follow-up, and data collection | 7&8 | **Study site selection**  A school from the flooded area was selected, and the KAP was compared with unflooded area. Based on these criteria and by using a convenience sampling, two schools were selected as study sites: 1) Mahmud Mahyiddin Secondary School, Pasir Pekan, Tumpat (flooded area) which experienced a recent dengue outbreak and affected by flood; and 2) Kubang Kerian Secondary School, Kubang Kerian which had recent dengue outbreak and not affected by flood (unflooded area).  **Study instruments**  A standardized questionnaire (Additional file 1) was developed using a baseline from the previous questionnaires on related studies [20-22]. Emphasis was given on the age group that responded to this tool. The questionnaire was translated into Bahasa Malaysia (National language of Malaysia, Additional file 2) and was made sure that the original meaning was retained. The dengue awareness booklet was bilingual (English and Bahasa Malaysia) as well. The booklet covers knowledge on DF and *Aedes* mosquitoes, which mainly aimed to expose people on the seriousness of the disease, its spread, the vectors and their characteristics, prevention and control measures to eliminate breeding grounds and prevention of mosquito bites. |
| Participants | 6 | (*a*) *Cohort study*—Give the eligibility criteria, and the sources and methods of selection of participants. Describe methods of follow-up  *Case-control study*—Give the eligibility criteria, and the sources and methods of case ascertainment and control selection. Give the rationale for the choice of cases and controls  *Cross-sectional study*—Give the eligibility criteria, and the sources and methods of selection of participants | 7 | The study was performed among 203 secondary schoolchildren between the age of 13-17 years old. This population was chosen based on the idea explained by Beinner et al. [18] that the youngsters within this age range are able to involve in abstract cognitive thinking and plan theory. This enables them to transform their thinking into systematic practical solutions based on rationale. Another essential point is that at this age level, the perception of environmental awareness extends beyond common place limits. In addition, it is expected that teenagers at this age serve as young educators as they tend to share their knowledge and experiences with their family and also to other community members. |
|  |  | (*b*) *Cohort study*—For matched studies, give matching criteria and number of exposed and unexposed  *Case-control study*—For matched studies, give matching criteria and the number of controls per case |  |  |
| Variables | 7 | Clearly define all outcomes, exposures, predictors, potential confounders, and effect modifiers. Give diagnostic criteria, if applicable |  | Knowledge, Attitude and Practice scores in pre- and post-tests |
| Data sources/ measurement | 8* | For each variable of interest, give sources of data and details of methods of assessment (measurement). Describe comparability of assessment methods if there is more than one group | 9-10 | **Data collection**  After obtaining consent from the volunteers, a pre-test questionnaire was administered to them. The participants were allocated a reasonable time to finish answering the questionnaire. Overall, it took about 20-30 minutes for them to complete the questionnaire. Shortly after the pre-test, the booklet was distributed to each respondent. The respondents were given a health education which started with a 15 minutes lecture using the booklet. In order to ensure the effective dissemination of knowledge among the respondents, interactive sessions such as discussions, briefing and question and answer (Q&A) were included as well. At the end of the session, the respondents were requested to share the messages and booklet they obtained through the study to family and friends. In addition, the respondents were also provided with small gifts with dengue educational messages to further stimulate changes in dengue preventive practices. The same set of questionnaire was re-administered (post-test) to the same group of respondents after a week. The respondents were again allocated reasonable amount of time to answer the questionnaire to allow them to recall memory. The pre- and post- tests were not linked to preserving confidentiality. Only respondents that participated in the pre-test were allowed to complete the post-test. There was a total of 208 students participated in the pre-test and 168 students in the post-test, from both schools. |
| Bias | 9 | Describe any efforts to address potential sources of bias  11 |  | The data of the completed questionnaires were entered into Microsoft Excel program and double-checked before analysis. |
| Study size | 10 | Explain how the study size was arrived at 8 |  | **Sample size**  The estimation of the minimum required sample size was calculated using Rao Soft® online sample size calculator [19]. Since the KAP level of the study population is unknown, we assumed the most statistically conservative response distribution is possible at 50%. To obtain a representative sample of the schoolchildren (<900 student population in both schools) with 90% confidence interval and 8% margin of error, we estimated a minimum of 95 students required from each school. A total of 108 students from flooded and 95 students from unflooded areas participated as volunteers in this study. |

Continued on next page

| Quantitative variables | 11 | Explain how quantitative variables were handled in the analyses. If applicable, describe which groupings were chosen and why | 10-11 | The knowledge scores varied from 0 – 13 points. Bloom’s cutoff point (60-80%) [24] was used to classify the scores into three levels: high level (80-100%) with scores between 11 – 13, moderate level (60-79%) with scores between 8 – 10 and low level (less than 59%) with scores between 0 – 7. Attitude section contained 11 positive and negative statements which were rated by using Likert’s scale [25]. For positive statement scores, the rating scale was measured as: strongly agree – 5, agree – 4, neither agree or disagree – 3, disagree – 2 and strongly disagree – 1. For negative statements, the scores were opposite: strongly agree – 1, agree – 2, neither agree or disagree – 3, disagree – 4 and strongly disagree – 5. The individual scores varied from 11 to 55. Responses were summed up, and means were calculated. The scores were categorized into three levels: Positive attitude (44– 55), Neutral attitude (38-43) and Negative attitude (11-37) using Bloom’s cut off point. The responses for practice were assessed as zero-one indicator (dummy) variables. The variables were given one for “yes” and zero for “no”. The classification was based on: Good practice (8 – 9 scores), Fair practice (6 – 7) and Poor practice (0 – 5). (Equal to Bloom’s cut off point, 60 – 80%). The section on practices after the flood to avoid dengue infection was assessed by using dummy variables: one for “yes” and zero for “no” based on: Good practice (4 – 6 scores), Fair practice (2 – 3) and Poor practice (0 – 1). (Equal to Bloom’s cut off point, 60 – 80%). |
| --- | --- | --- | --- | --- |
| Statistical methods | 12 | (*a*) Describe all statistical methods, including those used to control for confounding | 11-12 | **Data analysis**  The data of the completed questionnaires were entered into Microsoft Excel program and double-checked before analysis. The analysis of the data was performed in Statistical Package for Social Sciences (SPSS) version 22. Data were checked for normality (Shapiro-Wilk) prior to analysis, and natural log-transformation (ln[y+1]) was made due to the homogeneity of variance. The socio-demographic characteristics of the study population were divided based on the flooded and unflooded areas. Descriptive statistics in terms of frequency (n), percentage (%), mean and standard deviation (SD) was used to express the data. For analytical statistics, Independent T-test was used to compare between pre- and post-tests results and also to compare the baseline information between these two study sites. The correlation coefficient was used to describe the correlation between knowledge-attitude, knowledge-practice and attitude-practice. Multinomial logistic regression was carried out using JMP statistical package version 13 to identify factors associated with high level of dengue prevention practices. The factors were socio-demographic, knowledge (high, moderate, low) and attitude (positive, neutral, negative) level. A stepwise procedure with backward method was used to create the best fit model for the factors mentioned above by choosing the most significant values, and it was run separately for flooded and unflooded areas. Least significant factors (*P*>0.05) were excluded from the equation to obtain the best fit reliable equation for the model. |
|  |  | (*b*) Describe any methods used to examine subgroups and interactions |  | n/a |
|  |  | (*c*) Explain how missing data were addressed |  | n/a |
|  |  | (*d*) *Cohort study*—If applicable, explain how loss to follow-up was addressed  *Case-control study*—If applicable, explain how matching of cases and controls was addressed  *Cross-sectional study*—If applicable, describe analytical methods taking account of sampling strategy |  | n/a |
|  |  | (*e*) Describe any sensitivity analyses |  | n/a |
| Results | | | | |
| Participants | 13* | (a) Report numbers of individuals at each stage of study—eg numbers potentially eligible, examined for eligibility, confirmed eligible, included in the study, completing follow-up, and analysed | 12 | Table 1 shows the socio-demographic characteristics of the 203 respondents according to the study site. Among the 203 respondents, 53.2% were students from the flooded area, and 46.8% were from the unflooded area. |
|  |  | (b) Give reasons for non-participation at each stage | 12 | However, not all respondents from all the age groups were able to participate in the survey due to school activities and exams during the survey period. |
|  |  | (c) Consider use of a flow diagram |  | n/a |
| Descriptive data | 14* | (a) Give characteristics of study participants (eg demographic, clinical, social) and information on exposures and potential confounders | 12 | The age of the respondents ranged between 13 to 17 years old; majority belonged to the age group of 14 years (39.4%). However, not all respondents from all the age groups were able to participate in the survey due to school activities and exams during the survey period. Of the study respondents, 57.1% were female and 42.9% were male. The common monthly family income ranged between MYR 900-1500 (MYR: Malaysian Ringgit) (53.7%) in both study sites. Most of the respondents live in Bungalow/Village/Flats type of house (80.8%) and 56.2% of the respondents stated that their house is surrounded by a moderate level of vegetation. There were about 54.7% of the total respondents stated that there is a moderate level of mosquito density in their neighborhood, while only 4.9% of the respondents stated severe mosquito density with estimation of more than 100 mosquitoes. Most of the respondents from Mahmud Mahyiddin Secondary School, which we considered as flooded area, were flood victims (76.8%) at their residential area, whereas respondents of Kubang Kerian Secondary School, which we considered as unflooded area, showed a lesser number of flood victims (23.2%) at their residential area, (*P*<0.05). There were 29 (14.3%) households in total had the history of DF in the year of 2015. There was no significant difference in fogging frequency between both study sites (*P*>0.05). It can be concluded from the respondents’ feedback that fogging is carried out once every more than two months. |
|  |  | (b) Indicate number of participants with missing data for each variable of interest |  | n/a |
|  |  | (c) *Cohort study*—Summarise follow-up time (eg, average and total amount) |  | n/a |
| Outcome data | 15* | *Cohort study*—Report numbers of outcome events or summary measures over time | 14,17,22 | **Knowledge on dengue**  Table 2 shows the distribution of knowledge level on dengue, its spread, the vectors and the symptoms. Both study sites showed an increase in respondents with “high knowledge” and a decrease in “moderate and low knowledge” from pre- to post-test. The flooded area showed higher increases in the percentage of respondents with “high knowledge” (12.1%) as compared to respondents in the unflooded area (2.3%) (Table 2). There were no respondents reported as “low knowledge” in both study sites in the post-test.  **Attitude towards dengue prevention.**  In this section, the students answered 11 questions which can give a total score of 55. Distribution of attitude level towards dengue prevention is shown in Table 4. Both study sites showed the same trend, whereby there was an increase in the positive attitude, decrease in the neutral attitude and a slightly or very minimal increase in the negative attitude, which might be due to unequal sample sizes during pre- and post-tests. The unflooded area showed higher increases in respondents with “positive attitude” (10.4%) as compared to respondents in the flooded area (5.8%) (Table 4).  **Practices regarding dengue prevention**  Table 6 shows that majority of the respondents from both the study sites showed an increment in good practice level from pre- to post-test especially those from the flooded area (17.1%). Respondents from the flooded area showed a more significant increase in practice from pre- to post-test for question no. 3, 5 and 6, whereas respondents from the unflooded area only showed a significant increase in question 9 (*P*<0.05) (Table 7). However, it can be concluded that most of the respondents already have a good practice level, even at the pre-test level with their scores for each question were mostly more than 90%, except for question 6, which states that wearing light colored clothes to prevent mosquito bites. Thus, it indicates that the respondents were less aware of this preventive measure and the health education booklet has helped them to improve their practice level towards dengue prevention by 21%. |
|  |  | *Case-control study—*Report numbers in each exposure category, or summary measures of exposure |  |  |
|  |  | *Cross-sectional study—*Report numbers of outcome events or summary measures |  |  |
| Main results | 16 | (*a*) Give unadjusted estimates and, if applicable, confounder-adjusted estimates and their precision (eg, 95% confidence interval). Make clear which confounders were adjusted for and why they were included |  | n/a |
|  |  | (*b*) Report category boundaries when continuous variables were categorized |  | n/a |
|  |  | (*c*) If relevant, consider translating estimates of relative risk into absolute risk for a meaningful time period | 27 | Table 11 and 12 shows multinomial regression analysis of the associations between socio-demographic variables, knowledge and attitude levels on the practice (based on our conceptual framework in Fig 1). Presence of mosquitoes (mosquito density) in neighborhoods was more likely to have high dengue preventive practices in the flooded area (*P*<0.05). Respondents with the high and moderate level of knowledge and attitude were more likely to have high practice levels in the flooded area (*P*<0.05). In both locations, age and dengue history were common factors that influence the high dengue preventive practice levels (*P*<0.05). Gender, income, house type and flood victim were not significantly associated with higher level of dengue prevention practice in the flooded area, whereas, in the unflooded area, the non-significant associates were flooded victim, vegetation and attitude level (*P*>0.05). A best fit model was created by using the most significant factors as predictors for high practice level (Table 13). |

Continued on next page

| Other analyses | 17 | Report other analyses done—eg analyses of subgroups and interactions, and sensitivity analyses | 25-26 | **Correlation between knowledge, attitude and practice scores.**  The correlation between KAP scores revealed a significant positive correlation between knowledge-attitude for the flooded area and knowledge-practice for the unflooded area (*P*<0.05). No significant correlation was observed between attitude and practice in both locations. The degree of correlation was found to be low (r_s_<0.5) for both locations (Table 10).  **Overall comparisons between variables**  Overall, all of the criteria tested showed a numerical increase between pre- and post-test for both study sites. However, the significant increase was only observed in certain criteria only: School in the flooded area showed a significant increase in mean knowledge and practice (*P*<0.05) whereas school in the unflooded area showed a significant increase in mean knowledge only (*P*<0.05) (Table 9). The comparison of KAP means scores using Independent T-test between flooded and unflooded area revealed that students from the unflooded area had relatively higher levels of knowledge (*P*<0.05) as compared to students from the flooded area (Table 9). Insignificant differences were observed for attitude and practice criteria between these two areas. |
| --- | --- | --- | --- | --- |
| Discussion | | | | |
| Key results | 18 | Summarise key results with reference to study objectives | 29-30 | The study showed a significant increase in knowledge scores from pre- to post-test in both study sites. This shows that the booklet was able to increase their knowledge on dengue, its vectors and other important information through the health education program. The method of the survey conducted can be viewed as an educational method. The briefing, discussion and Q&A session were also conducted during delivery of the booklet. The study is in agreement with other health educational studies that knowledge increased when the study population was supplied with the health educational materials [14, 21, 26, 27].  The respondents did not show a significant increase of attitude scores after the health education. A similar observation was obtained by Lennon and Coombs [28] when assessing attitudes-beliefs change after conducting dengue educational board game. They rationalized their finding with limitation in their study that it only involves one treatment which is the board game that they used as a tool for dengue hemorrhagic fever health education. The changing in attitudes and beliefs related to health requires multiple treatments, processes and a longer period [29]. Since our study also involves only one-time health education, it is quite difficult to expect for sudden changes in attitudes in a very short period.  Regarding the dengue prevention practices, the mean scores increased in both study sites but significant increase was observed in the flooded area. More than 90% of the respondents stated that they performed practices such as rearing mosquito larvivorous fish in tanks/pools, turn over containers to avoid water collection, clearing up bushes/vegetation and garbage around the house to eliminate mosquito breeding sites and use the mosquito bed nets to avoid mosquito bites. One of the reasons for the high score in practice compared to knowledge and attitude in this study is probably due to items listed under practice section were related to their routine activities in their house or school for controlling mosquito breeding grounds. Whereas, the content for knowledge and attitude sections were more specified and detailed on dengue and its vector. However, we could not determine in our study how all of these selected practices are actually translated into practices in reality. Based on the answers given, fewer respondents practiced wearing light-colored clothes to avoid mosquito bites. This indicates that they were less aware of the information that mosquitoes are attracted to dark colors compared to the bright colors [30]. A similar finding was reported among the Malaysian public by Wong et al. [21], which only one-third of the study participants reported that they wear bright colored clothing to avoid mosquito bites. |
| Limitations | 19 | Discuss limitations of the study, taking into account sources of potential bias or imprecision. Discuss both direction and magnitude of any potential bias | 33 | In addition, only those participants who took part in the pre-test were allowed to complete the post-test, thus the pre- and post-tests were not individually linked, and socio-demographic information was collected only during the pre-test. Thus, there might be possibilities that those who have completed the pre-test might have differed from those completed the post-test. |
| Interpretation | 20 | Give a cautious overall interpretation of results considering objectives, limitations, multiplicity of analyses, results from similar studies, and other relevant evidence | 33 | Despite for all that, our assessment and educational program were considered successful in assessing the KAP and to increase the knowledge in dengue among the study participants over a short period. |
| Generalisability | 21 | Discuss the generalisability (external validity) of the study results | 33-34 | Assessment of the KAP level of the schoolchildren revealed a high level of knowledge on dengue, neutral attitude and good level of practice. The dengue awareness health education program successfully improved students’ knowledge related to dengue in both schools. It is important to educate children from the root so that individuals with good KAP can be created. Such education programs should also be directed towards elder populations in the future to study their KAP related to dengue. The findings suggest the need to target dengue education programs to communities with lower dengue risks and mosquito densities where public discerns as a lower chance of getting dengue infection. |
| Other information | |  | | |
| Funding | 22 | Give the source of funding and the role of the funders for the present study and, if applicable, for the original study on which the present article is based | 34 |  |

*Give information separately for cases and controls in case-control studies and, if applicable, for exposed and unexposed groups in cohort and cross-sectional studies.

**Note:** An Explanation and Elaboration article discusses each checklist item and gives methodological background and published examples of transparent reporting. The STROBE checklist is best used in conjunction with this article (freely available on the Web sites of PLoS Medicine at http://www.plosmedicine.org/, Annals of Internal Medicine at http://www.annals.org/, and Epidemiology at http://www.epidem.com/). Information on the STROBE Initiative is available at www.strobe-statement.org.
