# Supplementary material for: School-based health education for dengue control in Kelantan, Malaysia: Impact on knowledge, attitude and practice
Source: PLoS Negl Trop Dis. 2020 Mar 27;14(3):e0008075. doi: 10.1371/journal.pntd.0008075 (PMC7141698; doi:10.1371/journal.pntd.0008075)
Supplement: S1 Appendix — (DOCX) [file pntd.0008075.s002.docx]

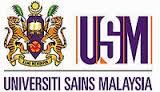


***QUESTIONNAIRE***

Thank you for participating in this research survey, which is conducted as a part of doctoral research study in School of Biological Sciences, Universiti Sains Malaysia, Pulau Pinang.

From this survey, we would like to investigate community knowledge on *Aedes* mosquitoes, prevention and control strategies associated with dengue fever. We specially targeting on secondary school students for this research.

Main purpose of this research is to educate public to create awareness on prevention on dengue cases and enhance knowledge to curb future dengue outbreak in Kelantan, especially after flood. A special educational booklet will be distributed to each student which provides information on *Aedes* mosquitoes and dengue prevention. Students will be required to complete a same set of questionnaire which will be given before and after distributing the education booklet.

Your views/answers are important to us. Please note that there is no right or wrong answers in this questionnaire. The questionnaire will last approximately 15 minutes.

Your responses and feedback obtained from this study will be kept confidential and will be used for research purposes only.

*Thank you very much for your participation and cooperation!*

**Dr. Wan Fatma Zuharah Wan Musthapa & Ahbi Rami Rattanam**

**PhD Researcher**

**School of Biological Sciences**

**Universiti Sains Malaysia**

**11800 Minden**

**Pulau Pinang**

**Email:** [**abbyrra88@yahoo.com**](mailto:abbyrra88@yahoo.com)

Please tick (✓) at your answers in provided space

**PART A: SOCIO-DEMOGRAPHIC CHARACTERISTICS**

1. Age (years) ___________
2. Gender ( ) Male ( ) Female
3. Monthly average household income ( ) Above RM 3001 ( ) RM 1501-3000

( ) RM 900- 1500 ( ) None

1. Education level­­­­­­­­­­­ ( ) Upper secondary ( ) Lower secondary
2. Type of house you’re living ( ) Bungalow/Village house ( ) Terrace/Twin house

( ) Condominium/Apartment/Flat

1. Did your house/neighborhood affected by flood in December 2014? ( ) Yes ( ) No
2. Does your house surrounded by many plants/trees/vegetation?

( ) Many ( ) Moderate ( ) Low ( ) None

1. Mosquito abundance in your neighborhood

( ) Severe ( ) Moderate ( ) Low ( ) None

1. Have you/ your family had dengue fever in this year? ( ) Yes ( ) No
2. Fogging frequency in your neighborhood

( ) Regularly ( ) At times ( ) Rarely ( ) Never

**PART B: KNOWLEDGE ON DENGUE, DENGUE SPREAD, VECTOR AND SYMPTOMS**

|  |  | **Yes** | **No** |
| --- | --- | --- | --- |
| 11. | Do you aware of dengue? |  |  |
| 12. | Dengue is a virus |  |  |
| 13. | Dengue is a serious illness |  |  |
| 14. | Dengue is transmitted to human by bites of infective mosquitoes |  |  |
| 15. | Human get infected by dengue by drinking dirty water |  |  |
| 16. | The two main vectors of dengue is *Aedes aegypti* and *Aedes albopictus* |  |  |
| 17. | Dengue patients will develop symptoms such as severe fever, headache, rashes, deep muscular and joint pain. |  |  |
| 18. | Mosquitoes that transmit dengue virus bite only during day |  |  |
| 19. | The mosquitoes that transmit dengue virus lay their eggs in dirty water |  |  |
| 20. | Stagnant water in empty containers, used tires, trash cans and flower pots can be possible breeding sites of mosquitoes. |  |  |
| 21. | There are proper vaccine/medications available for treatment of dengue |  |  |
| 22. | Only way to prevent dengue is by eliminating breeding grounds of dengue |  |  |
| 23. | Abate can be used to kill mosquito larvae in water storages |  |  |

**PART C: ATTITUDE TOWARDS PREVENTION OF DENGUE FEVER**

|  |  | **Strongly**  **Disagree** | **Disagree** | **Neither Agree nor Disagree** | **Agree** | **Strongly Agree** |
| --- | --- | --- | --- | --- | --- | --- |
| 24. | Are you at risk of getting dengue fever? |  |  |  |  |  |
| 25. | Dengue fever can be cured. |  |  |  |  |  |
| 26. | Dengue patient needs immediate treatment and hospitalization |  |  |  |  |  |
| 27. | It is possible to recover from dengue fever by eating paracetamol |  |  |  |  |  |
| 28. | Eradication of mosquito breeding ground is responsibility of public health authorities and volunteers |  |  |  |  |  |
| 29. | Elimination of larval breeding ground should be conducted every 1-2 times/year |  |  |  |  |  |
| 30. | Only fogging is enough to control mosquito population |  |  |  |  |  |
| 31. | Healthy person will not get dengue infection |  |  |  |  |  |
| 32. | Sleeping in mosquito/bed net will prevent mosquito bites and dengue infection |  |  |  |  |  |
| 33. | You will allow health inspectors to conduct inspections for larval breeding sources inside/outside house |  |  |  |  |  |
| 34. | You play vital role to curb dengue fever in your surroundings. |  |  |  |  |  |

**PART C: PRACTICES AGAINST DENGUE INFECTION**

|  |  | **Yes** | **No** |
| --- | --- | --- | --- |
| 35. | Eliminate standing water around the house to eliminate mosquitoes |  |  |
| 36. | Rear mosquito eating fish in tanks/pools to reduce mosquitoes |  |  |
| 37. | Turn over/Cover tightly containers to avoid water collection |  |  |
| 38. | Use mosquito bed nets to avoid mosquito bites |  |  |
| 39. | Use insecticide to kill mosquitoes |  |  |
| 40. | Wear light colored and fully covered clothes to avoid mosquito bite |  |  |
| 41. | Clear up bushes/vegetation around house to reduce mosquitoes |  |  |
| 42. | Cleaning of garbage/trash around house |  |  |
| 43. | Government spray insecticide (fogging ) to kill mosquitoes |  |  |

**PART D: PRACTICES DURING FLOOD TO ACOID DENGUE INFECTION**

|  |  | Yes | No |
| --- | --- | --- | --- |
| 44. | Sleep inside mosquito impregnated nets |  |  |
| 45. | Use smoke/mosquito coil to drive away mosquitoes |  |  |
| 46. | Use insecticide spray/aerosol to kill mosquitoes |  |  |
| 47. | Stay indoor |  |  |
| 48. | Did not take any precautions |  |  |

**BAHAGIAN F: SOURCE OF INFORMATION OF DENGUE**

Please choose your source of information of dengue (you may choose more than one option)

( ) Newspaper ( ) Radio ( ) Television ( ) School

( ) Health professionals ( ) Pamphlets/Banner ( ) Family/Friends/Relatives

( ) Magazines ( ) Others
