# Supplementary material for: School-based health education for dengue control in Kelantan, Malaysia: Impact on knowledge, attitude and practice
Source: PLoS Negl Trop Dis. 2020 Mar 27;14(3):e0008075. doi: 10.1371/journal.pntd.0008075 (PMC7141698; doi:10.1371/journal.pntd.0008075)
Supplement: S2 Appendix — (DOCX) [file pntd.0008075.s003.docx]

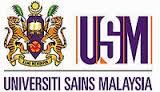


***SOAL SELIDIK***

Terima kasih kerana mengambil bahagian dalam kajian penyelidikan ini, yang dijalankan sebagai sebahagian daripada projek oleh Kementerian Pengajian Tinggi dan juga kajian penyelidikan kedoktoran di Pusat Pengajian Sains Kajihayat, Universiti Sains Malaysia, Pulau Pinang.

Dari kajian ini, kami ingin mendalami pengetahuan masyarakat mengenai nyamuk *Aedes*, pencegahan dan strategi kawalan yang berkaitan dengan demam denggi. Kami secara khasnya mensasarkan pelajar sekolah menengah untuk kajian ini.

Tujuan utama kajian ini adalah untuk mendidik orang ramai bagi memupuk kesedaran mengenai pencegahan kes-kes demam denggi dan meningkatkan pengetahuan bagi membendung wabak denggi di masa depan di Kelantan, terutama selepas banjir. Sebuah buku kecil yang menyediakan maklumat tentang nyamuk *Aedes* dan pencegahan denggi akan diedarkan kepada setiap pelajar. Pelajar akan dikehendaki melengkapkan satu set soal selidik yang akan diedarkan sebelum dan selepas pengedaran buku kecil “Dengue Awareness Educational Kit”.

Pandangan / jawapan anda adalah penting bagi kami. Sila ambil perhatian bahawa tidak ada jawapan yang betul atau salah dalam soal selidik ini. Soal selidik ini akan mengambil masa selama lebih kurang 10-15 minit.

Jawapan dan maklum balas yang diperoleh daripada kajian ini anda adalah sulit dan akan digunakan untuk tujuan pengajian sahaja.

Terima kasih untuk penyertaan dan kerjasama anda!

**Dr. Wan Fatma Zuharah Wan Musthapa & Ahbi Rami Rattanam**

**Unit Penyelidikan Kawalan Vektor**

**Pusat Pengajian Sains Kajihayat**

**Universiti Sains Malaysia**

**11800 Minden**

**Pulau Pinang**

**E-mel:** [**abbyrra88@yahoo.com**](mailto:abbyrra88@yahoo.com)

Sila tandakan (✓) pada jawapan anda di ruang yang disediakan.

**BAHAGIAN A: CIRI-CIRI SOSIO-DEMOGRAFI**

1. Umur (tahun)__________
2. Jantina ( ) Lelaki ( ) Perempuan
3. Purata pendapatan bulanan isi rumah ( ) Lebih RM 3001 ( ) RM 1501-3000

( ) RM 900- 1500 ( ) Kurang daripada RM 900

1. Tahap pendidikan ( ) Menengah atas ( ) Menengah rendah
2. Jenis rumah yang anda didiami ( ) Rumah banglo/kampung ( ) Rumah teres/berkembar ( ) Kondominium/Apartmen/Pangsapuri
3. Adakah rumah/kediaman anda ditimpa banjir pada Disember 2014 ( ) Ya ( ) Tidak
4. Adakan rumah anda dikelilingi oleh banyak tumbuh-tumbuhan/pokok?

( ) Banyak ( ) Sederhana ( ) Kurang ( ) Tiada

1. Ketumpatan nyamuk di kawasan kediaman anda

( ) Teruk (>100) ( ) Sederhana (50-100) ( ) Kurang (<50) ( ) Tiada

1. Adakah anda/keluarga anda dijangkiti demam denggi dalam tahun ini? ( ) Ya ( ) Tidak
2. Kebarangkalian semburan asap di kediaman anda

( ) Setiap minggu ( ) Setiap 2 minggu ( ) Setiap bulan ( ) Jarang (> 2 bulan) ( ) Tidak pernah

**BAHAGIAN B: PENGETAHUAN TENTANG DENGGI, PENYEBARAN DENGGI, VEKTOR DAN SIMPTOM**

|  |  | **Ya** | **Tidak** |
| --- | --- | --- | --- |
| 11. | Adakah anda sedar akan denggi? |  |  |
| 12. | Denggi ialah virus |  |  |
| 13. | Denggi ialah penyakit yang serius |  |  |
| 14. | Denggi ditransmisi kepada manusia oleh gigitan nyamuk yang terinfektif |  |  |
| 15. | Manusia dijangkiti oleh denggi dengan meminum air yang kotor |  |  |
| 16. | Dua vektor utama denggi ialah *Aedes aegypti* dan *Aedes albopictus* |  |  |
| 17. | Pesakit denggi akan mengalami simptom seperti demam yang teruk, sakit kepala, ruam, sakit otot dan sendi yang mendalam. |  |  |
| 18. | Nyamuk yang sebarkan virus denggi gigit semasa siang hari sahaja |  |  |
| 19. | Nyamuk yang sebarkan virus denggi bertelur dalam air yang kotor |  |  |
| 20. | Air di dalam bekas kosong, tayar terpakai, tin sampah dan pasu bunga boleh menjadi tempat pembiakan nyamuk |  |  |
| 21. | Terdapat vaksin/ubat-ubatan yang betul bagi rawatan denggi |  |  |
| 22. | Satu-satunya cara untuk mengelakkan denggi ialah melalui penghapusan tempat pembiakan nyamuk |  |  |

**BAHAGIAN C: SIKAP TERHADAP PENCEGAHAN DEMAM DENGGI**

|  |  | Sangat tidak setuju | Tidak setuju | Sama ada setuju atau tidak setuju | Setuju | Sangat setuju |
| --- | --- | --- | --- | --- | --- | --- |
| 24. | Adakah anda dalam risiko mendapat demam denggi? |  |  |  |  |  |
| 25. | Denggi boleh disembuh |  |  |  |  |  |
| 26. | Pesakit denggi perlu rawatan dan hospitalisasi dengan segera |  |  |  |  |  |
| 27. | Ia adalah mungkin untuk pulih daripada denggi dengan memakan paracetamol |  |  |  |  |  |
| 28. | Pembasmian tempat pembiakan nyamuk adalah tanggungjawab pihak berkuasa kesihatan awam dan sukarelawan |  |  |  |  |  |
| 29. | Penghapusan tempat pembiakan nyamuk perlu dijalankan setiap 1-2 kali setahun |  |  |  |  |  |
| 30. | Semburan asap itu sendiri cukup untuk mengawal populasi nyamuk |  |  |  |  |  |
| 31. | Orang yang sihat tidak akan mendapat jangkitan denggi |  |  |  |  |  |
| 32. | Tidur di dalam kelambu nyamuk/katil akan mengelakkan gigitan nyamuk dan jangkitan denggi |  |  |  |  |  |
| 33. | Anda akan membenarkan inspektor kesihatan untuk menjalankan pemeriksaan sumber pembiakan jentik-jentik di dalam/luar rumah |  |  |  |  |  |
| 34. | Anda memainkan peranan penting untuk membendung demam denggi di persekitaran anda |  |  |  |  |  |

**BAHAGIAN D: AMALAN UNTUK ELAKKAN JANGKITAN DENGGI**

|  |  | **Yes** | **No** |
| --- | --- | --- | --- |
| 35. | Hapuskan air bertakung di keliling rumah anda untuk menghapuskan nyamuk |  |  |
| 36. | Bela ikan yang memakan nyamuk di tangki/kolam untuk mengurangkan nyamuk |  |  |
| 37. | Terbalikkan/Tutup dengan rapat bekas-bekas untuk mengelakkan pengumpulan air |  |  |
| 38. | Gunakan kelambu nyamuk untuk mengelakkan gigitan nyamuk |  |  |
| 39. | Gunakan insektisid untuk membunuh nyamuk |  |  |
| 40. | Pakai pakaian berwarna terang dan dilindungi sepenuhnya untuk mengelakkan gigitan nyamuk |  |  |
| 41. | Bersihkan semak/tumbuhan di keliling rumah untuk mengurangkan nyamuk |  |  |
| 42. | Bersihkan sampah-sarap di keliling rumah |  |  |
| 43. | Kerajaan sembur insektisid (semburan asap) untuk membunuh nyamuk |  |  |

**BAHAGIAN E: AMALAN SEMASA BANJIR UNTUK ELAKKAN JANGKITAN DENGGI**

|  |  | Yes | No |
| --- | --- | --- | --- |
| 44. | Tidur di dalam kelambu impregnasi nyamuk |  |  |
| 45. | Guna asap/gegelung nyamuk untuk menghalau nyamuk |  |  |
| 46. | Guna insektisid semburan/aerosol untuk membunuh nyamuk |  |  |
| 47. | Tinggal di dalam rumah/bangunan |  |  |
| 48. | Tidak mengambil apa-apa langkah berjaga-jaga |  |  |

**BAHAGIAN F: SUMBER INFORMASI DENGGI**

Pilih sumber informasi denggi anda (Anda boleh pilih lebih daripada satu jawapan)

( ) Surat khabar ( ) Radio ( ) Televisyen ( ) Sekolah

( ) Profesional kesihatan ( ) Pamplet/Banner ( ) Keluarga/Rakan/Saudara-mara ( ) Majalah ( ) Lain-lain
